# Transcriptomic response of the red tide dinoflagellate, *Karenia brevis*, to nitrogen and phosphorus depletion and addition

**DOI:** 10.1186/1471-2164-12-346

**Published:** 2011-07-05

**Authors:** Jeanine S Morey, Emily A Monroe, Amanda L Kinney, Marion Beal, Jillian G Johnson, Gary L Hitchcock, Frances M Van Dolah

**Affiliations:** 1 Marine Biotoxins Program, NOAA National Ocean Service, Center for Coastal Environmental Health and Biomolecular Research, 219 Fort Johnson Rd., Charleston, SC 29412, USA; 2 Rosenstiel School of Marine and Atmospheric Sciences, University of Miami, 4600 Rickenbacker Cswy., Miami, FL 33149, USA; 3 Center for Marine Biotechnology and Biomedicine, Scripps Institution of Oceanography, University of California, San Diego, 9500 Gilman Dr. La Jolla, CA 92093, USA

## Abstract

**Background:**

The role of coastal nutrient sources in the persistence of *Karenia brevis *red tides in coastal waters of Florida is a contentious issue that warrants investigation into the regulation of nutrient responses in this dinoflagellate. In other phytoplankton studied, nutrient status is reflected by the expression levels of N- and P-responsive gene transcripts. In dinoflagellates, however, many processes are regulated post-transcriptionally. All nuclear encoded gene transcripts studied to date possess a 5' *trans*-spliced leader (SL) sequence suggestive, based on the trypanosome model, of post-transcriptional regulation. The current study therefore sought to determine if the transcriptome of *K. brevis *is responsive to nitrogen and phosphorus and is informative of nutrient status.

**Results:**

Microarray analysis of N-depleted *K. brevis *cultures revealed an increase in the expression of transcripts involved in N-assimilation (nitrate and ammonium transporters, glutamine synthetases) relative to nutrient replete cells. In contrast, a transcriptional signal of P-starvation was not apparent despite evidence of P-starvation based on their rapid growth response to P-addition. To study transcriptome responses to nutrient addition, the limiting nutrient was added to depleted cells and changes in global gene expression were assessed over the first 48 hours following nutrient addition. Both N- and P-addition resulted in significant changes in approximately 4% of genes on the microarray, using a significance cutoff of 1.7-fold and p ≤ 10^-4^. By far, the earliest responding genes were dominated in both nutrient treatments by pentatricopeptide repeat (PPR) proteins, which increased in expression up to 3-fold by 1 h following nutrient addition. PPR proteins are nuclear encoded proteins involved in chloroplast and mitochondria RNA processing. Correspondingly, other functions enriched in response to both nutrients were photosystem and ribosomal genes.

**Conclusions:**

Microarray analysis provided transcriptomic evidence for N- but not P-limitation in *K. brevis*. Transcriptomic responses to the addition of either N or P suggest a concerted program leading to the reactivation of chloroplast functions. Even the earliest responding PPR protein transcripts possess a 5' SL sequence that suggests post-transcriptional control. Given the current state of knowledge of dinoflagellate gene regulation, it is currently unclear how these rapid changes in such transcript levels are achieved.

## Background

Blooms of the dinoflagellate *Karenia brevis *occur nearly annually on the west Florida shelf, initiating in the late summer/early fall at depth before being transported along sub-surface currents and upwelled into coastal waters. There they may become further aggregated to form dense blooms that can dominate the water column for several months [1-3]. In recent years the duration and intensity of these blooms appear to have increased. A record red tide in 2005-6 persisted for more than 12 months and caused extensive mortality at all trophic levels in more than 2000 km^2 ^off the west central Florida coast [4]. In addition, the occurrence of blooms in the northern Gulf of Mexico in the past decade surpassed previous records, resulting in three major marine mammal mortality events in the Florida panhandle during that time period [5]. The recent intensity of bloom activity has raised alarm regarding the source of nutrients supporting high biomass blooms for extended durations, particularly whether anthropogenic sources of nitrogen and phosphorus due to extensive coastal development and agriculture have contributed to this increase [6]. Understanding the contribution of nutrient sources to the persistence of coastal blooms requires knowledge of how nutrient utilization by *K. brevis *permits this slow growing dinoflagellate to successfully compete in coastal waters.

*Karenia brevis *blooms experience diverse nutrient environments as they are transported shoreward from their near-bottom initiation in oligotrophic waters and become upwelled into surface coastal waters. *Karenia brevis *can efficiently utilize both inorganic and organic forms of carbon, nitrogen and phosphorus and its ability to carry out both autotrophic and heterotrophic metabolism is believed to provide a competitive advantage. This coupled with its low light adaptation may enable *K. brevis *to outcompete obligate autotrophs under low light conditions at depths found on the mid-Florida shelf [7]. Recent studies suggest that *K. brevis *is capable of accessing nitrogen from the sediment pore water through nutrient-directed swimming behavior [8]. Upwelling conditions on the west Florida shelf typically favor nitrate-assimilating diatoms, yet once initiated *K. brevis *often dominates hundreds of square kilometers and can represent a significant portion of the primary production [2,9,10]. Estimates of both N and P required to support dense blooms of *K. brevis *exceed the concentrations of either inorganic N and P available, which are typically 0.02-0.2 μM and 0.025-0.24 μM, respectively [10]. In contrast, organic N ranges from 8-14 μM and organic P from 0.2-0.5 μM. Current evidence suggests that N and P from multiple sources are required to maintain dense blooms, and that these sources vary temporally and spatially over the course of a bloom, including estuarine flux, atmospheric deposition, benthic flux, zooplankton excretion, and regenerated N released from *Trichodesmium *blooms and decomposing fish that result from bloom toxicity associated with brevetoxins [10,11].

The biochemical pathways by which *K. brevis *acquires and assimilates different sources of N and P are poorly characterized. However, the molecular characterization of these pathways in other phytoplankton groups provides some insight, particularly with the recent sequencing of three different species of diatom [12]. N-uptake is generally mediated by high affinity nitrate transporters and ammonium transporters. NO_3_^- ^is reduced by cytosolic nitrate reductase to NH_4_^+ ^and NO_2_^- ^[13]. NO_2_^- ^is reduced by nitrite reductase to NH_4_^+^. NH_4_^+ ^is assimilated in the plastid by glutamine synthetase II. A cytosolic glutamine synthetase, GSIII, acts separately to catalyze the assimilation of ammonium originating from the environment or cytoplasmic reactions. A number of genes in the N-assimilatory pathway in diatoms that are differentially regulated by the presence of NO_3_^- ^or NH_4_^+ ^have been identified as useful biomarkers for N-status, including glutamine synthetase II, nitrate reductase, and ammonium transporters [14]. Understanding the regulation of N-assimilation pathways in *K. brevis *may similarly provide insight into its utilization of nutrients during high density blooms.

Genomic studies have shed light on the mechanisms of phosphorus acquisition primarily in prokaryotic phytoplankton. In the cyanobacteria, *Synechocystis *[15], *Prochlorococcus*, [16], and *Synechococcus *[17], genes comprising the phosphorus responsive Pho regulon are strongly induced under P-starvation. These generally include a P-responsive histidine kinase phoR, a master regulator phoB, P-specific ABC transporters, and alkaline phosphatase phoA, as well as P-metabolism genes. However, the gene topology and even presence of P-responsive gene clusters may vary between ecotypes within a species, which may reflect their adaptation to different P regimes [18,19]. In the green algae, *Chlamydomonas reinhardtii*, a phosphorus starvation response (PSR1) transcription factor regulates inducible phosphate uptake mechanisms, including high affinity H^+^/P_i _symporters, Na^+^/P_i _cotransporters, and alkaline phosphatase [20]. Among other eukaryotic microalgae, P-transport and assimilation are less well characterized. An inducible high affinity phosphate transporter in the prasinophyte, *Tetraselmis chui*, was suggested to serve as a probe for monitoring phosphate stress [21]. Sequencing of the genome of the diatom *Thallassiosira pseudonanna *has also identified high affinity phosphate transporters [13]. The coccolithophore, *Emiliania huxleyi*, has a putative phosphate-repressible permease [22], which is up-regulated under low-P conditions [23]. Organic phosphate sources can be accessed in a wide variety of phytoplankton through the activity of extracellular alkaline phosphatases. Alkaline phosphatase transcripts in the coccolithophore *E. huxleyi *respond rapidly to fluctuations in phosphorus levels and are up-regulated in P-starved cells [24].

In contrast to other phytoplankton studied, it is currently unclear the extent to which dinoflagellates regulate gene expression at the level of transcription. Dinoflagellate chromatin lacks nucleosomes and exists in a tightly packed liquid crystal state, with peripheral arches of exposed DNA thought to be areas undergoing active transcription [25]. Many genes are present in multiple, divergent copies, often arrayed in tandem repeats. No recognizable promoter sequences (e.g., TATA boxes or initiator elements) have been identified in dinoflagellate genes. Instead, many physiological processes appear to be regulated at the translational level, including bioluminescence [26,27], carbon fixation [28], photosynthesis [29] and the cell cycle [30,31]. The discovery of a conserved 22-nucleotide *trans*-spliced leader sequence (SL) on diverse (possibly all) dinoflagellate transcripts [32,33] provides a possible mechanism to explain the prevalence of post-transcriptional gene expression. SL *trans*-splicing was first identified in trypanosomes, which carry out constitutive transcription of polycistronic messages. Polycistronic pre-mRNAs are processed into mature single-gene messages through the *trans*-splicing of the leader sequence at a splice acceptor site approximately 100 nt upstream of each start codon [34,35]. These *trans*-spliced messages then serve as a stable pool available for translation on demand. *Trans*-splicing is one of many examples of convergent evolution in dinoflagellates and trypanosomes [36] and, by analogy with trypanosomes, the widespread presence of the SL on dinoflagellate mRNAs suggests post-transcriptional control of gene expression. Like trypanosomes, the intergenic sequences between tandemly arrayed copies of actin genes in *Amphidinium carterae *possess splice acceptor sites approximately 100 nt upstream of each open reading frame [37]. Unlike trypanosomes, in which polycistronic messages contain a series of different genes, the few examples studied in dinoflagellates consist of tandemly arrayed copies of the same gene [37]. In some cases, genes present in tandem arrays lack stop codons and are therefore not only co-transcribed, but also co-translated into polyproteins that are matured by protein cleavage [38-40]. Nonetheless, the presence of the SL mechanism does not preclude the possibility of differential rates of transcription. For example, major diurnal differences in transcript abundance of peridinin chlorophyll a proteins and light harvesting complex proteins in *Amphidinium carterae *correlate with DNA methylation within or near their coding regions, suggesting that their differential transcription may be regulated by differential chromatin condensation [41].

In the current study we sought to determine how nutrient limited *K. brevis *responds to nutrient (N, P) addition and in particular if its response includes a response at the transcriptome level. To assess transcriptome response we utilized a *K. brevis *11K feature microarray to first compare transcript profiles in cultures under nutrient replete conditions and in N- or P-starved cells. We then carried out N- or P-additions and compared the transcript profiles over 48 hours following nutrient addition. Following nutrient addition, differential transcript profiles were observed as early as 1 hour. The earliest responding transcripts were dominated in both nutrient treatments by nuclear-encoded transcripts for PPR proteins, which are involved in chloroplast and mitochondria RNA processing, as well as photosystem and ribosomal genes, suggesting a reawakening of the cellular metabolic machinery.

## Methods

### *K. brevis *Culture Conditions

Batch cultures of *K. brevis *(Wilson isolate) were maintained in 1 L bottles in *f*/2 medium using 20 μm filtered, autoclaved natural seawater (36 ‰) with the following modifications: ferric sequestrene was substituted for EDTA · Na_2 _and FeCl_3 _·6H_2_O and 0.01 μM (final concentration) selenous acid was added. The concentration of nitrate or phosphate in nutrient replete cultures was 883 μM or 36 μM, respectively. Nitrogen-limited cultures were adapted to 10 μM nitrate and phosphorus-limited cultures were adapted to 0.1 μM phosphate by a minimum of six serial log phase (Day 7) transfers prior to experimental treatments. All cultures were acclimated to a 16:8 h light:dark cycle at 25 ± 1°C and approximately 175-215 μmol photons·m^-2^·sec^-1 ^illumination from cool white lights.

### Nutrient Addition Studies

For nutrient addition studies, triplicate nutrient replete and low nutrient 1 L cultures (10 μM NO_3 _or 0.1 μM PO_4_) were grown to stationary phase. Using sodium nitrate or sodium phosphate, 155 μM NO_3 _or 168 μM PO_4 _were added to stationary phase cultures. Nutrient replete cultures and N- or P-limited cultures were harvested at the time of nutrient addition (Time 0; n = 3) and total RNA extracted. Following nitrogen addition, triplicate cultures were harvested at 4, 12, 24, and 48 h post-addition and total RNA extracted. For phosphorus addition, triplicate cultures were harvested at 1, 4, 24, and 48 h post-phosphorus addition and total RNA extracted. All time points occurred during the light phase in order to avoid potential diurnal effects on gene expression [31]. Growth curves were established in triplicate parallel 1 L cultures by collecting 5 mL of nutrient replete, N- or P-limited, and N- or P-supplemented cells every two days, fixing in glutaraldehyde, and counting using a Beckman Coulter Multisizer 3 (Fullerton, CA). The specific growth rate (K') was calculated for each of the culture conditions [42].

### RNA Processing

At each time point post-nutrient addition, triplicate one liter cultures were harvested by centrifugation at 600 × *g *for 10 m and total RNA was extracted using Tri-Reagent according to the manufacturer's protocol (Molecular Research Center, Inc., Cincinnati, OH). RNA was resusupended in nuclease-free water and further processed using an RNeasy mini column with on-column DNase digestion (Qiagen, Valencia, CA) according to manufacturer's protocol. RNA was then quantified using a NanoDrop ND-1000 (Wilmington, DE) and qualified on an Agilent 2100 Bioanalyzer (Santa Clara, CA). RNA was also extracted from nutrient replete and nutrient deplete cultures (n = 3) at the time of nutrient addition.

### Microarray Analysis

A *K. brevis *oligonucleotide microarray containing 10,263 60-mer probes designed from our cDNA library as described by Lidie *et al. *[43] was used for these studies, using a one-color protocol. Total RNA (600 ng) was amplified and labeled with Cy3 dye using a low input linear amplification kit (Agilent, Santa Clara, CA). The amplified, labeled RNA was quantified using a NanoDrop ND-1000 and 480 ng of Cy3 labeled targets were hybridized to the array for 17 hours at 60°C. After hybridization, arrays were washed according to the manufacturer's protocol. Microarrays were imaged using an Agilent microarray scanner. Images were extracted with Agilent Feature Extraction version 9.5.3.1 and data analyzed with Rosetta Resolver version 7.2 gene expression analysis system (Rosetta Biosoftware, Cambridge, MA). Using a rank consistency filter, features were subjected to a combination linear and LOWESS normalization algorithm. Based on the Rosetta error model designed for the Agilent platform, a composite array was generated at each time point from triplicate arrays (representing three biological replicates), in which the data for each feature underwent a normalization, intensity averaging, and error estimation based on data from the replicate arrays making up the composite [44]. The composite arrays were then used to build ratios at each time point, relative to nutrient deplete cultures the time of nutrient addition, and a trend analysis was used to determine the expression pattern of genes throughout the time course. Only features with absolute differential expression of 1.7 fold or greater and a p-value ≤ 10^-4 ^on the composite array in at least one time point were included in trend analyses. Since genes that operate within a pathway are often coordinately regulated, these data were then clustered using a Euclidean metric by a K-means clustering algorithm to discern subsets of genes with similar expression patterns. The trend set was further analyzed for enrichment of specific gene ontology (GO) categories using the modified Fisher's Exact test in Blast2GO version 2.3.6 [45].

### Quantitative Real-Time PCR

Differentially expressed genes of interest were selected for validation of the microarray results by quantitative real-time PCR (qPCR). Triplicate reverse transcription reactions were carried out using 200 ng total RNA with an oligo(dT) primer using Ambion's RETROscript Kit (Austin, TX). Primer pairs specific for the contig of interest were designed and (Additional File 1) used for qPCR on an ABI 7500 using the ABI Power SYBR Green master mix (Applied Biosystems, Foster City, CA). The optimal annealing temperature for each primer set was determined prior to the analysis of experimental samples. The specificity of each primer set and size of the amplicon were verified by analysis with an Agilent Bioanalyzer 2100 and further confirmed by melt curve analysis. The efficiency of each primer set was determined using a standard curve of cDNA from *K. brevis*. A cycle threshold (C_t_) was assigned at the beginning of the logarithmic phase of PCR amplification and the difference in the C_t _values of the control and experimental samples were used to determine the relative expression of the gene in each sample. Contig_5157, a cyclin dependent kinase, or Contig_2004, a hypothetical protein, were used for normalization of the nitrogen- or phosphorus addition study, respectively, with the ΔΔC_t _method [46] as their expression did not change significantly in microarray or qPCR experiments (ANOVA, p > 0.05). Correlation to the N-addition microarray data set was determined by Pearson Product-Moment Correlation while correlations to the P-addition data set were determined by Spearman's Rho, due to a non-normal data distribution, using JMP version 5.1.2 (SAS Institute, Cary, NC).

### Amplification of trans-spliced messages

Reverse transcription of total RNA was carried out using Ambion's RetroScript kit with an oligo(dT) primer according to manufacturer's instructions. To confirm the presence of the spliced leader sequence on PPR transcripts, a truncated SL primer (5'-TCCGTAGCCATTTTGGCTC-3') was used in combination with gene-specific primers as previously described in [32]. Briefly, amplification was carried out for 25 cycles at an annealing temperature of 60°C using Qiagen's HotStarTaq Master Mix and the resulting PCR products were qualified on an Agilent Bioanalyzer 2100, purified using a Qiagen PCR purification kit, and cloned using Invitrogen's TOPO TA for Sequencing prior to sequencing in both directions using M13 forward and reverse primers on an ABI Prism 3730xl sequencer by SeqWright (Houston, TX).

## Results

### *K. brevis *Growth Behavior Under Different Nitrogen Regimes

**Figure 1:**
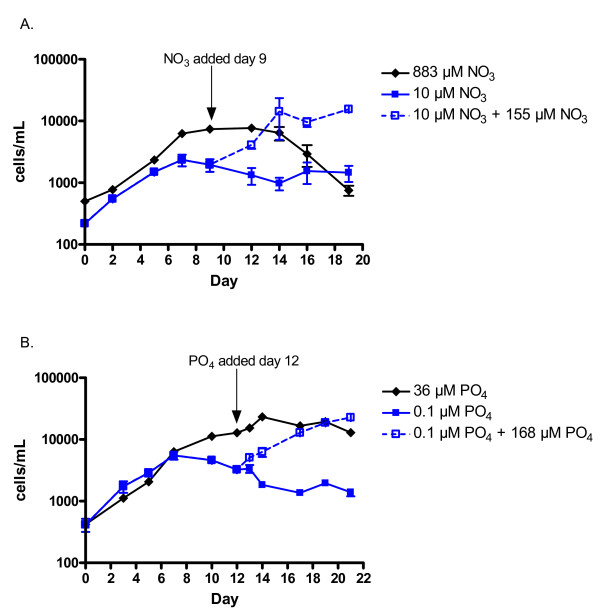
**The growth of *K. brevis *under different nutrient treatments**. Growth of *K. brevis *cultures under *f*/2, 883 μM NO_3_, and N-deplete, 10 μM NO_3_, conditions and their response to 155 μM NO_3 _addition during stationary phase (day 9) (A). Growth of *K. brevis *cultures under *f*/2, 36 μM PO_4_, and P-deplete, 0.1 μM PO_4_, conditions and their response to 168 μM PO_4 _addition during stationary phase (day 12) (B). Data are mean ± S.E.M. of cell concentrations in 6 replicate cultures prior to N- or P-addition and 3 replicate cultures post-addition (where 3 of 6 cultures received nutrient addition, 3 remained untreated).

*Karenia brevis *cultures grown in *f*/2 medium with a starting cell concentration of ~500 cells · mL^-1 ^underwent approximately 7 days of logarithmic growth at a division rate of 0.6 div · day^-1 ^(Figure 1A). Cultures grown in 10 μM NO_3 _had a shorter logarithmic growth phase of approximately 5 days, entering stationary phase at a lower cell concentration (Figure 1A) and with a somewhat lower division rate of 0.48 div · day^-1^.

When 155 μM nitrate was added to N-depleted cultures once they reached stationary phase (day 9), measurable growth was observed within 3 days of N-addition (Figure 1A). In contrast, cultures grown in *f*/2 did not exhibit significant growth following addition of NO_3 _on day 9 (data not shown). These results indicate that the cultures grown in 10 μM NO_3 _entered stationary phase early because of N-depletion.

### Transcriptomic Evidence for N-depletion

Microarray analysis was first used to compare the transcriptomes of cultures grown in *f*/2 (n = 3) to cultures grown in 10 μM NO_3 _(n = 3) in stationary phase on day 9 to establish whether signatures of N-depletion were evident in the 10 μM NO_3 _cultures, given their rapid growth response to N-addition. Individual microarrays were hybridized with RNA from each of the triplicate cultures. The triplicate arrays were then used to generate an error-weighted composite array for *f*/2 or 10 μM NO_3 _day 9 cultures and the log ratio of fluorescence intensity was generated for each probe on the array. A 1.7-fold difference with a p-value ≤10^-4 ^was used as a significance cutoff based on our previous establishment of significance limits using these arrays [47]. Using this cutoff, 1102 probes (10.7% of array features) differed between *f*/2 and 10 μM NO_3 _stationary phase cultures, 454 of which are annotated. No significant enrichment for specific gene ontologies was found within these features. Among the annotated features, there was little evidence of hallmark signs of N-depletion in the 10 μM NO_3 _cultures relative to the *f*/2 cultures on Day 9 (Table 1). Data mining of microarrays from a separate study of gene expression in *K. brevis *over a complete growth curve in *f*/2 media (Johnson JG, Morey JS, Neely MG, Ryan JC, Van Dolah FM: Transcriptome remodeling associated with chronological aging in the dinoflagellate *Karenia brevis*, submitted) showed increases in expression of some nitrogen assimilation genes as cultures moved from log phase to stationary phase (Table 1), while a comparison of the *f*/2 log phase cultures to the 10 μM NO_3 _stationary phase cultures in the current study showed consistent signs of N-depletion, indicated by significant up-regulation of type III glutamine synthetases, nitrate/nitrite transporters, and an ammonium transporter (Table 1). Together with the differential growth responses to NO_3 _addition these data suggest that *K. brevis *grown in 10 μM NO_3 _were N-depleted once entering stationary phase.

**Table 1:** Fold-change in expression of genes in the nitrogen assimilation pathway in cultures grown in 10 μM NO_3 _vs *f*/2 medium.

			**Stationary 10 μM NO**_**3**_**/** Stationary *f* /2		Stationary *f*/2/Log *f*/2		**Stationary 10 μM NO**_**3**_**/** Log *f*/2	
			
Gene	Contig #	Top BLASTx e-value	fold change	p-value	fold change	p-value	fold change	p-value
Ammonium transporter	11443	1.00E-54	1.30	0.0023	**2.00**	8.58E-16	**2.50**	9.14E-13
								
Glutamine synthetase, type III	214	1.00E-16	1.00	0.9574	**2.69**	5.68E-10	**2.58**	6.58E-07
	2216	1.00E-07	**2.34**	1.00E-07	-1.05	0.4424	**2.07**	1.95E-06
	2215	1.00E-15	1.93	0.0007	1.08	0.1709	**1.98**	0.0002
type I or II	2193	5.00E-58	1.12	0.3888	1.28	0.1693	1.44	0.0337
	547	1.00E-19	-1.28	0.0071	1.29	0.0005	1.00	0.9323
								
Nitrate transporter, NRT2	780	2.00E-45	1.00	0.5756	1.95	0.0002	1.77	0.0006
	3886	1.00E-11	1.31	0.2593	1.97	0.1347	2.68	0.01
	2750	4.00E-22	1.39	0.0249	-1.68	5.68E-07	-1.20	0.0347
	77	2.00E-32	-1.15	0.2534	-1.25	2.28E-05	-1.36	0.0317
								
Nitrate/nitrite transporter	1567	1.00E-98	1.21	0.401	**3.26**	5.80E-13	**3.70**	2.68E-09
	11115	2.00E-99	1.25	0.1016	**1.70**	1.48E-06	**2.18**	0.6118

Bolded values meet the significance cutoff of ±1.7 and p ≤ 10^-4^.

### Transcriptomic Response of N-depleted *K. brevis* to Nitrogen Addition

**Figure 2:**
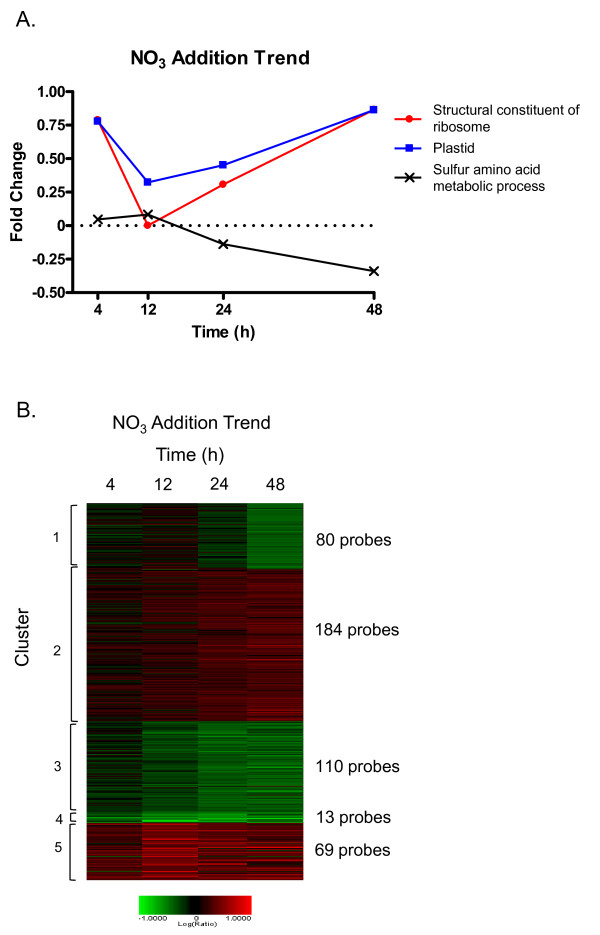
**Transcriptomic response to nitrate addition**. Selected significantly enriched GO terms within the N-addition trend set relative to all sequences on the array (A) using a modified Fisher's Exact Test in Blast2GO (FDR < 0.05). The plotted values are the average fold change for all probes in the enrichment group. Heat map of *K. brevis *trend set for N-addition (B) microarray studies. K-means clustering with a Euclidean distance metric were applied to a filtered data set requiring at least 1.7 fold change and a p-value ≤ 0.0001 in at least one time point for inclusion.

Microarray analysis of gene expression was carried out on N-depleted *K. brevis *at 0, 4, 12, 24, and 48 h following the addition of 155 μM NO_3 _on day 9. Individual microarrays were hybridized with RNA from individual cultures at each time point (n = 3), which were then used to generate an error-weighted composite array at each time point. All raw gene expression data have been deposited in NCBI's Gene Expression Omnibus (GEO, http://www.ncbi.nlm.nih.gov/geo/, GEO series accession number GSE28362). For analysis of gene expression throughout the time course, a high quality trend set was compiled including only those features that exhibited at least 1.7 fold change and a p ≤ 0.0001 in at least one time point. This filtering resulted in a trend set of 456 features (4.4% of array features), of which 218 could be annotated (BLASTx e-value ≤ 1e^-4^; Additional File 2). Few features (39) qualified for the trend set at the 4 h time point whereas 307 qualified at the 48 h time point, which represented the greatest change relative to the N-depleted cultures at time of NO_3 _addition (Time 0). Of the nitrogen assimilation pathway components that were up-regulated in the N-depleted cells relative to N-replete cells, none changed significantly over the 48 h time course following N-addition. Overall, PPR proteins were the most highly represented gene family in the trend set, comprising 13.3% of the annotated features with significant change in expression. Furthermore, 32% of the features corresponding to PPR proteins on the microarray were included in the trend set. Enrichment analysis by Blast2GO indicated significant over-representation of several categories of genes in the trend set, including structural constituents of the ribosome, amino acid metabolic/biosynthetic processes, and plastid functions (Fisher's Exact, FDR < 0.05, Figure 2A). Whereas the overall trends for the ribosome and plastid functions were up-regulation, the overall expression levels of the sulfur amino acid metabolic processes decreased over the 48 h time course. Within the enriched GO term for sulfur amino acid metabolic processes, genes involved in catalysis of the transfer of a methyl group from S-adenosyl-methionine were up-regulated, whereas genes involved in methionine biosynthesis were down-regulated (Additional File 3).

Since genes that operate within a pathway are often coordinately regulated, the trend set was next analyzed using K-means clustering with a Euclidean distance metric to discern subsets of genes with similar expression patterns. Five gene clusters identified distinctly different patterns of expression following NO_3 _addition (Figure 2B).

Cluster 1 contained 80 features showing little change at 4 and 12 h, mild down-regulation at 24 h followed by stronger down-regulation at 48 h post-N-addition (Figure 2B). Thirty-four features were annotated and included 2 DnaJ chaperone proteins (Hsp 40), a Cu/Zn SOD, and all 4 of the serine/threonine protein phosphatases included in the trend set (Additional File 2). No significant enrichment of any GO term was found in this cluster. These genes were not up-regulated in N-deplete cultures relative to log or stationary phase cultures grown in *f*/2 (data not shown). Therefore, this strong down-regulation does not appear to be not a return to steady state levels over this short time course.

Cluster 2 contained 184 features showing little change at 4 and 12 h with increasing up-regulation through 24 and 48 h (Figure 2B). Among the 87 annotated features were several ribosomal proteins, PPR proteins, photosystem 1 (psI) and photosystem 2 (psII) proteins, and several enzymes involved in DNA repair (Additional File 2). As in Cluster 1, no significant enrichment of any GO term was observed.

**Figure 3:**
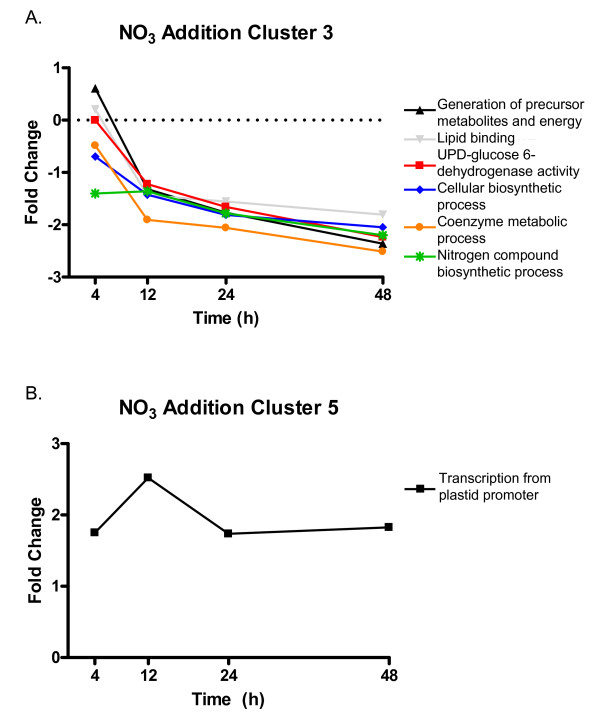
**Significantly enriched GO categories in response to N-addition in *K. brevis***. Selected categories of enrichment from cluster 3 (A) and cluster 5 (B) of the N-addition trend set. Enrichment was determined relative to all sequences on the array using a modified Fisher's Exact Test in Blast2GO (FDR < 0.05). The plotted values are the average fold change for all probes in the enrichment group. Clusters 1, 2, and 4 of the trend set did not have any significant enrichment of GO terms.

Cluster 3 included 110 probes, 55 of which were annotated (Additional File 2). These features show little change at 4 h post-N-addition with stronger down-regulation throughout the remainder of the time course (Figure 2B). Many genes involved in various aspects of amino acid metabolic processes were found in this cluster. As a result, this cluster shows significant enrichment of nitrogen compound biosynthetic processes as well as sulfate assimilation and reduction, and amino acid biosynthetic/metabolic processes (Fisher's Exact test, FDR < 0.05, Figure 3A). These enrichments are driven by the presence of 3-phosphoadenosine-5-phosphosulfate reductase and homoserine dehydrogenase genes involved in methionine and threonine biosynthesis, which are down-regulated 2-3 fold. An ornithine carbamoyltransferase, involved in arginine biosynthesis, also belongs to the nitrogen compound biosynthetic processes term (Additional File 3).

Cluster 4 was a small cluster of only 13 features that show strong down-regulation throughout the time course (Figure 2B). Among the 6 features that were annotated there is no apparent commonality in function or localization (Additional File 2). Calreticulin, which qualified for the trend analysis at all 4 time points, was included in this cluster as was ubiquitin.

Cluster 5 contained 69 features that were strongly up-regulated throughout the time course, particularly at 12 h (Figure 2B). Of the 36 annotated features, 60% were PPR proteins (Additional File 2). Other genes in this cluster were helicases and genes involved in amino acid or ribosome binding. Together these created significant enrichment in GO categories corresponding to regulation of plastid transcription and plastid functions/organization (Fisher's Exact Test, FDR < 0.05, Figure 3B).

### qPCR Validation of the NO_3_ Addition Microarray Results

**Figure 4:**
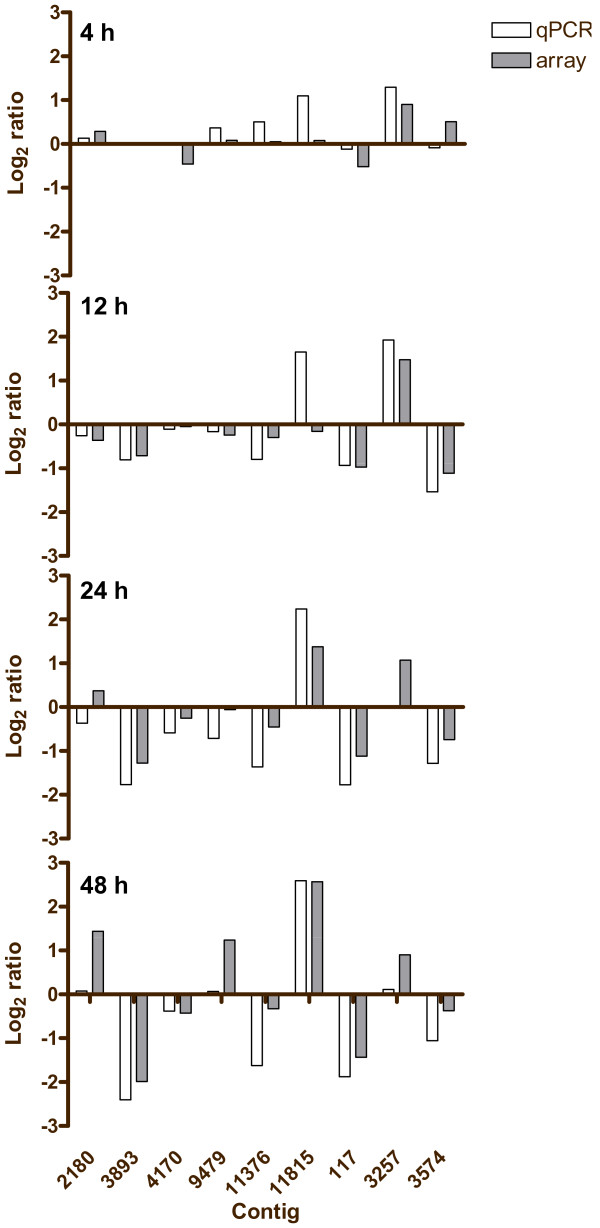
**Validation of the N-addition microarray study by qPCR**. Ten genes, including one normalizer, not shown, were selected for verification by qPCR. Overall, a correlation of 0.82 was observed (Pearson's, p < 0.0001, n = 36). The correlations at the 4, 12, 24, and 48 h time points were 0.56 (p = 0.113, n = 9), 0.84 (p = 0.005, n = 9), 0.91 (p = 0.0006, n = 9), 0.94 (p = 0.0002, n = 9), respectively.

Ten features were selected for verification by real-time PCR (Figure 4), including Contig_5041 a cdc2-like protein kinase, used for normalization with the ΔΔC_t _method. Changes in gene expression measured by qPCR strongly support the microarray results, with a correlation of 0.82 across the time series (p < 0.0001, n = 36). Correlations at individual time points increased throughout the time series, as did the magnitude of change observed. Disagreement between qPCR and microarray occurred only when minimal changes in expression were observed, as has been previously reported [47]. A minimal correlation of 0.56 (p = 0.113, n = 9) was observed at 4 h where, with one exception, all changes were less than ± 1.5 fold by both qPCR and array. Correlations increased to 0.84 at 12 h (p = 0.005, n = 9), 0.91 at 24 h (p = 0.0006, n = 9), and reached a maximum of 0.94 at 48 h (p = 0.0002, n = 9).

### *K. brevis *Growth Behavior Under Different Phosphorus Regimes

*K. brevis *cultures grown in *f*/2 carried out logarithmic growth for approximately 10 days, with a division rate of 0.56 div · day^-1 ^(Figure 1B). The cultures grown in 0.1 μM PO_4 _entered stationary phase after approximately 7 days, and had a similar division rate of 0.53 div · day^-1 ^(Figure 1B). Consequently, nutrient addition was carried out on day 12, when both P-deplete and cells grown in *f*/2 were in stationary phase. When 168 μM PO_4 _was added to the *f*/2 cultures on day 12 no additional growth was seen (data not shown). In contrast, the P-depleted cultures resumed growth following PO_4 _addition, exceeding the maximal cell density of cultures grown in *f*/2 by day 21 (Figure 1B). The growth response to PO_4 _addition in the P-depleted cells suggests that they entered stationary phase at least in part due to P-depletion

### Transcriptomic Evidence for P-depletion is Largely Absent

Microarray analysis was next used to compare the transcriptomes of cultures grown in *f*/2 (n = 3) to cultures grown in 0.1 μM PO_4 _(n = 3) on day 12 in stationary phase in order to establish whether any signatures of P-limitation were evident in the P-depleted cultures. Using the 1.7 fold and p ≤ 0.0001 cutoffs, 1259 probes (12% of array features) differed significantly, 548 of which are annotated. Comparison of the annotated features provided little indication of differences in P-status, with mixed responses of acid phosphatases being the only observable difference (Table 2). Data mining of microarrays from a study of gene expression in *K. brevis *grown in *f*/2 media over a growth curve (Johnson JG, Morey JS, Neely MG, Ryan JC, Van Dolah FM: Transcriptome remodeling associated with chronological aging in the dinoflagellate *Karenia brevis*, submitted) showed an increase in expression reported by two probes for plastid inorganic pyrophosphatase and purple acid phosphatase in stationary phase relative to log phase cultures (Table 2). Only one of these plastid inorganic pyrophosphatase probes was increased in the P-starved cells on day 12 relative to log phase cells in *f*/2, as was purple acid phosphatase. Vacuolar type H+ translocating inorganic pyrophosphatases showed mixed response and two probes for type III glutamine synthetase were up-regulated 1.8-2.4 fold in the P-depleted stationary phase cells relative to P-replete log phase cultures (Table 2). Alkaline phosphatase did not change in expression under any condition. Thus, although the rapid growth response following the addition of PO_4 _indicates P-starvation in these cells, the transcriptional profile was not informative of P-starvation based on these phosphorus transport and metabolism genes.

**Table 2:** Fold-change in expression of genes in the phosphorus assimilation pathway in cultures grown in 0.1 μM PO_4 _vs *f*/2 medium.


			**Stationary 0.1 μM PO**_**4**_**/** **Stationary *f*/2**		**Stationary *f*/2/Log *f*/2**		**Stationary 0.1 μM PO**_**4**_**/** **Log *f*/2**	
			
**Gene**	**Contig #**	**Top BLASTx** **e-value**	**fold change**	**p-value**	**fold change**	**p-value**	**fold change**	**p-value**

Mn-dependent inorganic pyrophosphatase	3803	1.00E-42	1.04	0.7064	-1.38	0.0002	-1.34	1.40E-05
								
Plastid inorganic pyrophosphatase	4129	1.00E-49	-1.42	4.24E-08	**1.76**	1.65E-23	1.25	0.0022
	4809	1.00E-103	1.16	0.2	**3.10**	8.52E-25	**3.41**	6.77E-21
								
V type H^+^-translocating inorganic pyrophosphatase	2166	3.00E-34	1.05	0.6177	1.33	0.0005	1.36	1.08E-06
	4595	1.00E-61	1.60	0.0001	-1.03	0.8259	1.50	0.0006
	7738	9.00E-40	1.31	0.0018	1.14	0.2264	1.45	0.0005
	8774	1.00E-24	-1.23	0.0366	-1.56	1.58E-06	**-1.92**	1.86E-07
	10241	9.00E-29	1.33	0.0019	1.46	0.0001	**1.92**	2.60E-08
								
Acid phosphatase	9839	2.00E-57	**1.80**	7.01E-09	-1.63	0.0081	1.15	0.3335
	4279	5.00E-31	**1.69**	6.52E-12	-1.31	2.64E-05	1.23	2.35E-05
	8626	1.00E-05	**-1.79**	2.80E-11	1.19	0.0211	-1.49	1.76E-11
	10776	1.00E-12	1.33	0.0108	1.02	0.7163	-1.28	0.0167
								
Purple acid phosphatase	10336	9.00E-20	-1.13	0.5343	**2.22**	0.0372	**2.03**	0.0952
								
Alkaline phosphatase	9702	9.00E-22	-1.1	0.3349	1.08	0.292	-1.01	0.9311
								
Plastid phosphate translocator	9723	1.00E-19	-1.20	0.0066	1.54	1.78E-10	1.28	4.26E-07
								
Glutamine synthetase, type III	214	5.00E-31	1.03	0.7576	**2.39**	4.14E-08	**2.42**	3.74E-10
	2215	2.00E-14	1.16	8.66E-06	1.09	0.2455	**1.75**	3.87E-07
	2216	5.00E-08	1.02	0.7891	-1.38	0.4855	-1.05	0.5808
type I or II	547	1.00E-19	-1.33	8.47E-05	1.29	3.85E-07	-1.09	0.1286
	2193	2.00E-58	1.29	0.0407	1.20	0.2996	1.51	0.0157

Bolded values meet the significance cutoff of ±1.7 and p ≤ 10^-4^.

### Transcriptomic Response of P-depleted *K. brevis *to Phosphorus Addition

Because we observed a surprisingly rapid change in the transcriptome as early as 4 h in the NO_3 _addition study, particularly among the PPR-repeat dominated cluster 5, we added an earlier time point of 1 h in the PO_4 _addition study. All raw gene expression data have been deposited in NCBI's Gene Expression Omnibus (GEO, http://www.ncbi.nlm.nih.gov/geo/, GEO series accession number GSE28419). A high quality trend set was compiled from 3 arrays representing biological replicates at 1, 4, 24, and 48 h following P-addition that included features that exhibited at least 1.7 fold change and a p ≤ 0.0001 in at least one time point. This filtering resulted in 425 features (4.1% of array features), of which 183 had BLASTx hits with an e-value ≤ 1e^-4 ^(Additional File 4). We did not see change in expression of any P-assimilation genes during the first 48 h following P-addition (Additional File 4). Very few features (23) were differentially expressed at the 1 h or 4 h time points (44), whereas 148 or 298 qualified for the trend set at 24 h or 48 h time point, respectively. Only 2 features qualified for inclusion in the trend set at all 4 time points, both of which were PPR containing proteins. PPR proteins were highly represented in the phosphate-responsive trend set showing, in general, maximal up-regulation at 4 h with continued elevated expression throughout the time course. Twenty-seven (14.8%) of the annotated features in the trend set were PPR proteins, representing 30% of all features corresponding to PPR proteins on the microarray.

**Figure 5:**
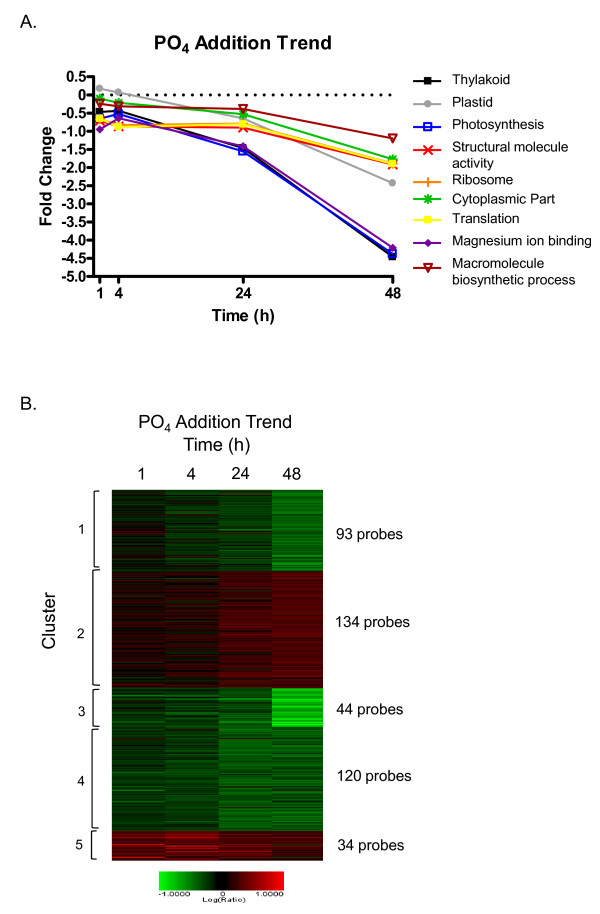
**Transcriptomic response to phosphate addition**. Selected significantly enriched GO terms within the P-addition trend set relative to all sequences on the array (A) using a modified Fisher's Exact Test in Blast2GO (FDR < 0.05). The plotted values are the average fold change for all probes in the enrichment group. Heat map of *K. brevis *trend set for P-addition (B) microarray studies. K-means clustering with a Euclidean distance metric were applied to a filtered data set requiring at least 1.7 fold change and a p-value ≤ 0.0001 in at least one time point for inclusion.

Blast2GO analyses indicated enrichment of the trend set relative to the array in several categories including structural constituents of the ribosome, rRNA binding, the plastid, and components of photosynthesis and electron transfer (Fisher's Exact, FDR < 0.05, Figure 5A). The trend set was next clustered by K-means using a Euclidean distance metric to discern subsets of genes with similar expression patterns (Figure 5B).

**Figure 6:**
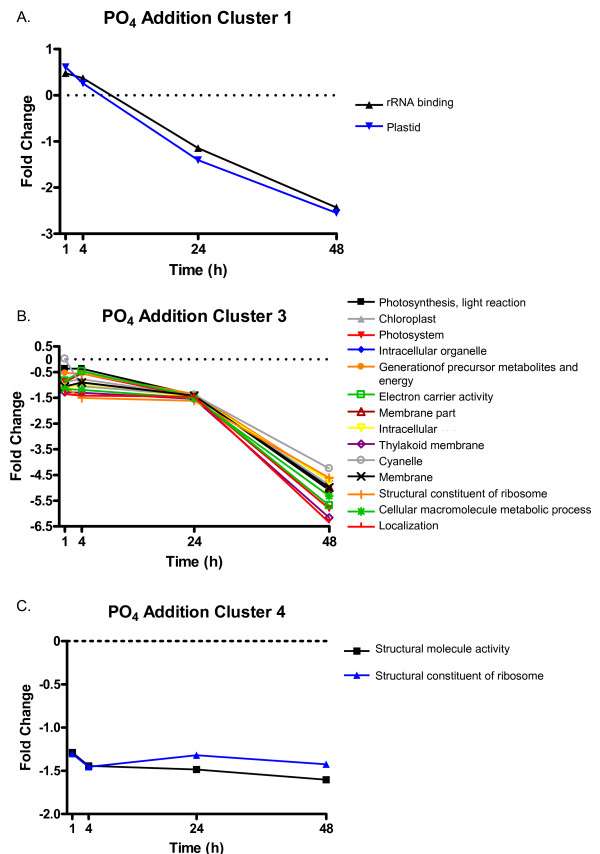
**Significantly enriched GO categories in response to P-addition in *K. brevis***. Selected categories of enrichment from cluster 1 (A), cluster 3 (B), and cluster 4 (C) of the P-addition trend set. Enrichment was determined relative to all sequences on the array using a modified Fisher's Exact Test in Blast2GO (FDR < 0.05). The plotted values are the average fold change for all probes in the enrichment group. Clusters 2 and 5 of the trend set did not have significant enrichment of any GO terms.

Cluster 1 contained 93 probes that showed little change at 1 h following P-addition and gradually decreased in expression over the remaining 48 h of the study (Figure 5B and Additional File 4). Among the 39 annotated features were ribosomal genes and photosynthesis-related genes. Enrichment analyses in Blast2GO indicated a significant enrichment of categories including rRNA binding and the chloroplast (Fisher's Exact test, FDR < 0.05, Figure 6A).

Cluster 2 contained 134 probes, 53 of which are annotated. These features showed gradually increasing expression throughout the time course following P-addition (Figure 5B). There was no significant enrichment of any GO category in the cluster. This cluster did contain several PPR proteins, RNA interacting proteins, and three polyketide synthases (Additional File 4).

Cluster 3 contained 44 probes that are down-regulated throughout the time course, with strong down-regulation at 48 h (Figure 5B and Additional File 4). Twenty-three of the features were annotated, of which most encode ribosomal proteins or photosystem proteins. Accordingly, Fisher's Exact test showed significant over-enrichment of many GO annotations, almost all of which are related to the chloroplast, photosynthesis, or ribosomes (FDR <0.05, Figure 6B). As these transcripts were not up-regulated in P-deplete cultures compared to either log or stationary phase cultures grown in *f*/2, the strong down-regulation does not appear to represent a return to typical P-replete levels during this time course.

Cluster 4 contained 120 features down-regulated at all time points, with mild down-regulation early and moderate down-regulation observed at 24 and 48 h (Figure 5B and Additional File 4). Among the 43 annotated features were several ribosomal proteins. Correspondingly, the Fisher's Exact test found that the structural constituent of the ribosome is significantly over-enriched (FDR <0.05, Figure 6C).

Cluster 5 was the smallest cluster of the trend with 34 features. These features are up-regulated throughout the time course, but showed maximal expression at 4 h post-P-addition (Figure 5B and Additional File 4). Among the 25 annotated features were 22 PPR proteins. One of the remaining 3 features in this cluster is an RNA-binding protein. Despite the prevalence of PPR proteins in this cluster, no significant enrichment was found. The lack of enrichment is most likely due to the incomplete annotation of many of these PPR proteins (i.e. many have not been assigned GO terms).

### qPCR Validation of the PO_4 _Addition Microarray Results

**Figure 7:**
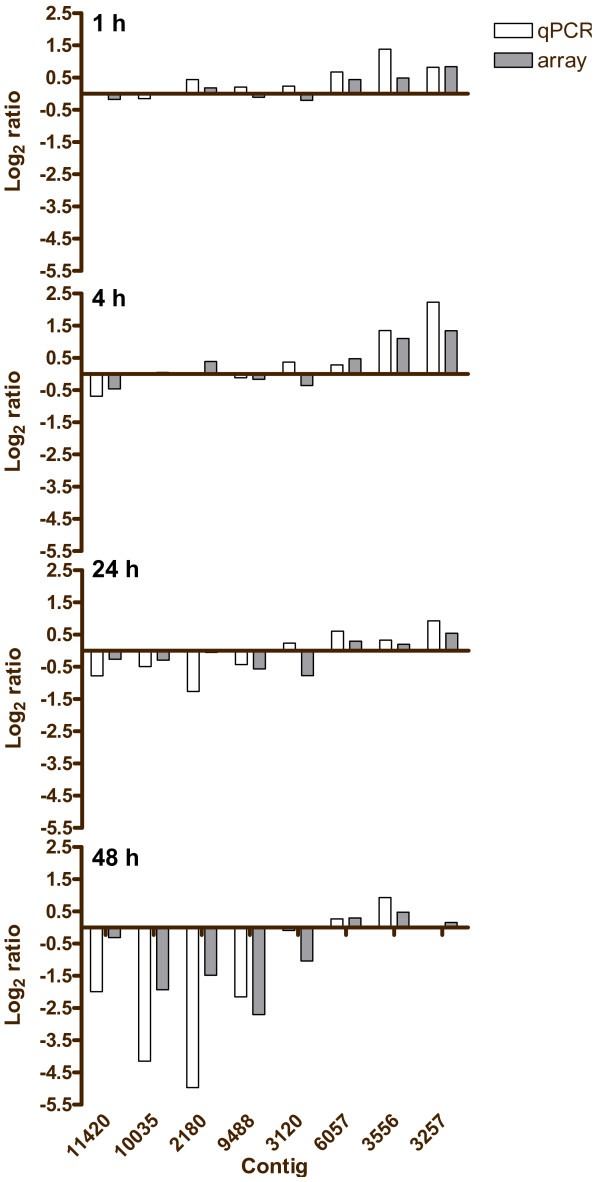
**Validation of the P-addition microarray study by qPCR**. Nine genes, including one normalizer, not shown, were selected for verification by qPCR. Overall, a correlation of 0.82 was observed (Spearman's Rho, p < 0.0001, n = 32). The correlations at the 1, 4, 24, and 48 h time points were 0.76 (p = 0.028, n = 8), 0.74 (p = 0.037, n = 8), 0.52 (p = 0.183, n = 8), 0.88 (p = 0.004, n = 8), respectively.

Nine features were selected for verification by real-time PCR (Figure 7), including Contig_2004 a hypothetical protein, used for normalization with the ΔΔC_t _method. Changes in genes expression measures by qPCR strongly supported the microarray results, with a correlation of 0.82 across the time series (p < 0.0001, n = 32). The strongest correlation of 0.88 (p = 0.004, n = 8) was observed at the 48 ht time point, which also exhibited the greatest changes of gene expression. Among the validated contigs, minimal changes were observed at 24 h post-addition and corresponded to the lowest correlation of 0.52 (p = 0.183, n = 8). The correlations at 1 h and 4 h post-addition were 0.76 (p = 0.028, n = 8) and 0.74 (p = 0.037, n = 8), respectively.

### Comparison of the Transcriptomic Response to Nitrogen or Phosphorus Addition

**Figure 8:**
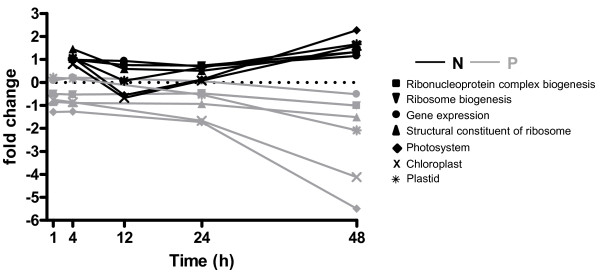
**Significantly enriched GO categories in response to both N- and P-addition in *K. brevis***. Enrichment was determined relative to all sequences on the array using a modified Fisher's Exact Test in Blast2GO (FDR < 0.05). The plotted values are the average fold change for all probes in the enrichment group.

The transcriptomes of NO_3_- or PO_4_-depleted cultures showed similar temporal responses to N- or P-addition, with limited changes in transcript levels observed prior to 12 h post-addition. The resulting trend sets were similar in size, with approximately 4% of array features responding to nutrient addition. Overall, the transcriptional changes measured in response to N-addition were of greater magnitude than those observed in response to P-addition. Eighty-two features, roughly 18% of features in the trend sets, were found in common between N- and P- addition (Additional Files 2 and 4). Of those found in common, the majority exhibited similar directions of response to N- or P-addition, including 21 PPR proteins. However, 12 features, including 30S and 60S ribosomal proteins and several photosystem proteins behaved oppositely in the two studies: in response to N-addition these features are up-regulated, whereas they are down-regulated following P-addition (Figure 8). The 82 features found in common between N- and P- addition are significantly enriched in GO categories corresponding to photosynthesis, the chloroplast, or ribosomes relative to the features included on the array (Fisher's Exact test, FDR < 0.05, Figure 8). The features unique to N-addition did not result in the significant enrichment of any GO category. In contrast, the features unique to P-addition that were significantly enriched were additional members of GO categories corresponding to the ribosome or rRNA binding, the chloroplast and photosynthesis (Fisher's Exact test, FDR < 0.05, data not shown).

### Early Responding Transcripts Possess the Spliced Leader

Given the presence of the spliced leader sequence on all dinoflagellate nuclear encoded transcripts investigated to date, and its implication of post-transcriptional control of gene expression, it was somewhat surprising to see the early response of gene transcripts in Cluster 5 of both the N- and P- addition experiments, which were dominated by PPR proteins. Some of these transcripts increased more than three-fold by 1 h following P-addition (e.g., probe numbers 183 and 3271, Additional File 4). This led us to question whether these early responding transcripts represent a class of genes not processed through the SL mechanism and under transcriptional control. Since the sequences from which probes on the array were designed are ESTs or contigs representing incomplete gene sequences, we selected representative early responding PPR containing transcripts for confirmation of a 5' SL sequence by performing PCR using a SL primer and gene-specific primers. Due to the high sequence similarity between PPR contigs in our *K. brevis *EST library, the reverse primers designed against contigs 183, 3257, 3556, and 3574, when paired with the SL primer, actually amplified multiple products. Thus, through cloning and sequencing we have identified the presence of the SL on just over 40 contigs annotated as PPR proteins. These results suggest that the early responding genes are not exceptions to the SL *trans*-splicing mechanism prevalent in dinoflagellates.

## Discussion

The absence of recognizable promoter sequences on dinoflagellate genes and the presence of 5' SL on dinoflagellate gene transcripts [32,33] suggest, by analogy with trypanosomes, the post-transcriptional control of gene expression. Consistent with this hypothesis, many physiological processes have been found to be regulated post-transcriptionally [26-31]. Similarly, microarray analysis of acute stress responses in *K. brevis *did not reveal the activation of stress genes [48] under conditions (1-4 hours) where they were shown to be induced at the protein level [49]. Microarray analyses found only ~3% of transcripts changing over a circadian day in *Pyrocystis lunula *[50] and *K. brevis *[31]. Yet in *K. brevis *~10% of transcripts were differentially expressed over a light-dark cycle [31], a percentage that does not differ substantially from other photosynthetic eukaryotes that utilize typical transcriptional controls [51,52]. Since microarrays report only changes in transcript abundance, it remains unresolved by what mechanism(s) these changes are achieved.

In this study, we explored in two different scenarios the response of the *K. brevis *transcriptome to nutrients. We first investigated the differences in the transcriptome between nutrient depleted cells and cells grown in nutrient replete media. This may be considered a chronic response in which the transcriptome has had an extended period of time to remodel, and therefore, changes in the transcriptome could result solely from differences in message stability. We found that the transcript profiles of N-depleted cells did indeed reflect their status by the increased expression levels of transcripts for N- acquisition. In contrast, evidence for P-limitation was not apparent from the transcriptome. We next investigated the acute response of the transcriptome of nutrient depleted cells to the addition of N or P over a short time course of 1-48 h following addition. The acute remodeling of the transcriptome within hours of nutrient addition was surprising, given the presence of the spliced leader on even the earliest responding transcripts. The resulting changes in transcript profile reflect primarily the rapid reactivation of the chloroplast metabolic machinery by nuclear encoded regulators. Changes in the expression of hallmark N- or P-acquisition genes were not observed upon addition of nitrogen or phosphorus to nutrient depleted cultures. To our knowledge there are currently no global transcriptional studies in phytoplankton following nutrient addition.

### Transcriptome Responses to Nitrogen Starvation and Addition

The increased expression of putative ammonium transporter, nitrate transporter and glutamine synthetase transcripts observed in N-starved *K. brevis *is consistent with their roles in N-uptake and assimilation. Transcript abundance of all three of these genes was strongly up-regulated in the N-depleted stationary phase cultures relative to nutrient replete cultures in log phase of growth. All of the up-regulated glutamine synthetases had homology to glutamine synthetase III (glnN), while other glutamine synthetase probes present on the array with homology to glutamine synthetase I or II did not respond. In the haptophyte *Isochrysis galbana *ammonium and nitrate transporters are strongly up-regulated under N-starvation, along with glutamine synthetase II as measured by qRT-PCR [53]. In contrast, glutamine synthetase II mRNA expression in the diatom *Skeletonema costatum *was low under N-starvation [54] and increased with NO_3 _addition following approximately a five day delay. Digital expression profiling of EST libraries of the diatoms *Phaeodactylum *and *Thallassiosira *revealed altered expression of numerous genes involved in nitrogen metabolism and regulatory elements in nitrogen starved cells [55]. Among these, an ammonium transporter was the most highly up-regulated gene in an N-starved *Phaeodactylum *library. Ammonium transporters have similarly been shown to increase in expression in N-starved diatom *Cylindrotheca *[56]. The nitrate reductase transcript in the diatom *Cylindrotheca *is expressed under N-starvation or in the presence of nitrate, but is inhibited by the presence of ammonium [57]. Thus it appears that the regulation of these pathways may differ somewhat across different phytoplankton phyla.

Following the addition of NO_3 _to N-depleted cells, we did not observe changes in the expression of any N-uptake or assimilation genes within the first 48 h. The responses in *K. brevis *were instead enriched in several processes including plastid functions, ribosomes, nitrogen compound metabolism, and amino acid biosynthesis. Members of these gene ontologies were also responsive in a microarray study of the *Arabidopsis *transcriptomic response at 2 and 24 h following N-addition [58]. In both studies, transcript levels for ribosome and plastid functions increased by 24 h. Confoundingly, transcripts belonging to the GO functions of nitrogen compound metabolism and amino acid biosynthesis differed in their direction of change, where their abundances increased in *Arabidopsis *by 24 h, but decreased in *K. brevis *over the 48 h time course. Specifically, in *K. brevis *genes involved in the synthesis of arginine, methionine, and threonine, such as homoserine dehydrogenase and ornithine carbamoyltransferase are down-regulated. These genes were not up-regulated under N-depletion so the observed down-regulation does not appear to be a return to levels expressed in N-replete log phase cultures. In contrast, genes involved in cysteine biosynthesis, including catechol-o-methyltransferase and cystathionine beta-synthase were increasingly up-regulated 1.76-1.90 fold starting at 12 h following N-addition. A possible explanation is that genes involved in the synthesis of amino acids exhibit an overall down-regulation during N-limitation. Upon addition of a N source biosynthesis of amino acids initiates and, as the sulfate needed to produce methionine is derived from cysteine [59], it is possible that the expression of genes involved in methionine biosynthesis will increase only after a sufficient pool of cysteine is produced as a result of increased nitrogen levels. The regulation of nitrogen and sulfur assimilation pathways has been shown to be tightly coupled in many other plants and algae including the green alga *Chlamydomonas reinhardtii *[60] with nitrogen limitation causing a down-regulation in genes involved in sulfate assimilation as observed in this study.

### Transcriptome Responses to Phosphorus Starvation and Addition

P-depleted cells showed little consistent indication of P-starvation through the transcript levels of genes putatively involved in P- uptake or utilization, despite the strong evidence based on the growth response to P-addition. Under P-stress, ATP pools are significantly reduced, affecting nearly all metabolic processes, including DNA, RNA, and phospholipid biosynthesis, as well as regulatory phosphorylation of proteins and generation of phosphorylated intermediates for photosynthetic carbon fixation. Plastid inorganic pyrophosphatases and plastid phosphate translocators are important mechanisms for recycling PP_i _needed for regenerating ATP used for CO_2 _fixation. We also queried acid phosphatases and vacuolar type H+-translocating inorganic pyrophosphatases, which in higher plants and *Chlamydomonas *increase in both expression and activity under P-starvation, thereby providing alternative energy sources to the limited ATP pools available under P-starved conditions [61]. These probes showed mixed responses to P-starvation in *K. brevis*. Lastly, alkaline phosphatase, whose activity is often used as an indicator of phosphate stress in phytoplankton, showed no response at the transcript level. Alkaline phosphatase enzyme activity has been shown to be induced in *K. brevis *under similar low phosphate (1 μM) conditions [62]. By comparison, in the coccolithophore *Emiliania huxleyi*, alkaline phosphatase transcripts are dramatically induced by phosphate starvation and rapidly repressed after phosphate addition [24]. The absence of any changes in transcript levels in the current study suggests this activity may be regulated at a translational or post-translational level, which is consistent with the presence of the SL mechanism.

Following P-addition, the transcriptome response was enriched in GO categories that include ribosome constituents, RNA binding, plastid, and electron transfer functions. As in the response to N-addition, the earliest changes were dominated by the increase in transcripts for PPR proteins that in the P study were measurable as early as 1 h following P-addition. However, in marked contrast with the response to N-addition, the ribosomal and chloroplast functions were strongly down-regulated by 24-48 h following P-addition. The reason for the disparity in response of these transcripts to N- and P-addition is unknown. It has been shown in yeast that the initiation of ribosome biogenesis is tied to a critical cell size that is controlled by nutrient signals [63]. While cell size was not measured in this study, N-limitation has been reported to decrease cell size while P-limitation increases cell size in other dinoflagellate and algal species [64,65]. Thus, the opposing responses of these genes may reflect complex differences in the physiological status of N- and P-starved cells that will require further investigation.

### PPR Proteins

The early responses of the transcriptome to both N and P were dominated by increases in transcripts for PPR proteins. Of the array features that responded significantly to nutrient addition, 29 and 25 annotated features of the N- and P- addition trend sets, respectively, were PPRs. This represents over 13% of annotated features in the trend sets, and approximately a quarter of the features annotated as PPRs on the array. PPR (pentatricopeptide repeat) proteins are a novel family of proteins first discovered when the *Arabidopsis *genome was sequenced, defined by a 35 amino acid (pentatricopeptide) motif that is repeated in tandem up to 30 times [66,67]. Most PPR transcripts in *Arabidopsis *possess chloroplast (~25%) or mitochondrial (~75%) targeting sequences [68]. PPR proteins are sequence-specific RNA binding proteins that, in plants, bind in a sequence-specific manner to unique organellar mRNAs, resulting in recruitment of enzyme complexes that modulate their expression through post-transcriptional processes, including editing, splicing, translation, and stability [67,69,70]. PPR proteins are absent from bacteria, but are present in all eukaryotes examined, generally at low copy number (5 and 6 in yeast and human, respectively [68]). *Trypanosoma brucei *is an exception among non-photosynthetic eukaryotes, encoding 28 unique PPR proteins [71,72] that are essential for mitochondrial rRNA biogenesis and stability [72]. Among the 11,000 unique genes in the *K. brevis *EST database approximately 100 are annotated as PPR proteins. Preliminary analysis of these PPRs contigs with ChloroP [73] has identified the presence of a chloroplast transit peptide on 40% of contigs from the EST database. Further, 100% of the contigs that have the 5' end (as defined by the presence of the splice leader) were found to have a chloroplast transit peptide in *K. brevis*.

The large representation of PPR proteins among the *K. brevis *transcripts responding to N- or P- addition was a driving force behind many of the enrichment categories involving the chloroplast and ribosomal proteins. Their peak expression (12-24 h following nutrient addition) generally preceded the changes in expression of the chloroplast encoded photosystem and electron transport genes, and ribosome and RNA binding proteins, which peaked at 48 h following N-addition. This temporal relationship, in addition to their known roles in organellar RNA processing, suggests a link between PPR transcript abundance and subsequent expression of chloroplast encoded genes.

### The Photosystem and Photosynthetic Electron Transport Chain

A limited number of plastid encoded genes involved in the photosynthetic electron transport chain are present on the array and were among the strongest responding transcripts to both N- and P-addition. The interpretation of chloroplast-encoded gene expression using the microarray requires special consideration because the oligo(dT) primed RNA labeling methods employed depend on the presence of a polyA tail, but in the chloroplast, polyadenylation serves as a signal for RNA degradation [74]. Therefore, an increase in transcript abundance of a chloroplast-encoded gene on the array may reflect increased polyadenylation, or destabilization of the message pool, rather than increased expression. Conversely, a decrease in chloroplast encoded transcripts on the array may reflect their decreased polyadenylation, or increased mRNA stability. At 48 h following N-addition, Photosystem I (p700 chlorophyll a apoproteins A1 and A2), photosystem II (44 kDa, 47 kDa, D1, and D2 proteins) and ATP synthase (CF0 α and F0 subunit C) transcripts were strongly up-regulated on the array, likely indicating increased turnover of these chloroplast encoded genes. Down-regulation (e.g., stabilization) of the same genes was observed as *K. brevis *moves from log phase into stationary phase (Johnson JG, Morey JS, Neely MG, Ryan JC, Van Dolah FM: Transcriptome remodeling associated with chronological aging in the dinoflagellate *Karenia brevis*, submitted), so the increase following nutrient addition may reflect the transition back to active cellular division.

Although pigment analyses were not performed, greening of the cells was clearly visible within 12 hours of N-addition as pigment production was re-established in chlorotic cells; this process preceded the increase in polyadenylated transcript abundance for photosystem and ATPase genes. Thus the observed increase in polyadenylated plastid messages late in the recovery from N-starvation may reflect a subsequent turnover of messages. Consistent with this interpretation, during greening of *Chlamydomonas reinhardtii *following N-addition an increase in photosystem transcripts occurred within 2-3 hours, then returned to basal levels within 12 hours [75]. In the current study, every one of the photosystem and ATP synthase transcripts that was up-regulated on the array following N-addition was down-regulated at 48 h following P-addition, possibly indicating increased message stability (i.e., decrease in polyadenylated messages). Although P-amended *K. brevis *cultures returned to growth with similar kinetics as those that received N-additions, the processes underlying this response clearly differ. No obvious greening of the cells occurred following P-addition as chlorosis did not occur in the P-limited cultures of *K. brevis*. In plants, both nitrogen and phosphorus limitations decrease photosynthesis but do so through different mechanisms. N-stress reduces photosynthesis directly by decreased light absorption capacity through diminished expression of photosystem protein complexes, whereas P-stress decreases rates of CO_2 _fixation through changes in the activity of Calvin cycle enzymes, both mechanisms resulting in further feedback inhibition of photosynthesis [76]. In *Chlamydomonas*, removal of either sulfate or phosphate reduces photosynthesis, but whereas S-depletion decreases chloroplast transcript levels, P-depletion results in an increase in chloroplast RNA stability and abundance [77]. The opposing responses of photosystem gene transcripts following the addition of N or P in the current study suggest differences also exist in the recovery from N- vs P-depletion at the level of chloroplast mRNA transcription and/or stability. A better understanding of this process will require targeted studies on *K. brevis *chloroplast regulation.

### Post-transcriptional Control in *K. brevis*

Given the prevalence of PPR proteins in these data sets and their rapid increase in response to nutrient addition, we were particularly interested in determining if these genes are among those under SL control, or if they represent a class of dinoflagellate genes regulated by transcription. Using PCR with a SL specific primer and a gene specific primer, we identified the presence of the 5' SL cap on more than 40 unique PPR gene transcripts. This suggests that the up-regulation of PPRs in response to nutrient addition does not reflect typical eukaryotic transcriptional control. Changes observed in a global transcriptional profile could be the result of several different mechanisms, including differential rates of *trans*-splicing, differential mRNA stability, and changes in chromatin structure that modulate transcription.

In trypanosomes, transcriptional silencing of the spliced leader gene (the only gene under transcriptional control) occurs in response to stress, leading to depletion of SL available for *trans*-splicing and the accumulation of polycistronic messages [78,79]. If this mechanism occurs in dinoflagellates, the recovery from nutrient starvation may be mediated by a rapid resumption in *trans*-splicing of polycistronic messages, which would generate an increased number of mature monocistronic mRNAs available for detection by the microarray without changes in transcription. However, gene expression in trypanosomes is further regulated by changes in RNA stability mediated by 3'UTR elements including U-rich instability elements (UREs), short interspersed degenerated retroposons (SIDERs), and RNA binding proteins [80]. A clock-controlled RNA-binding protein has been identified in *Chlamydomonas reinhardtii *(Chlamy 1) and is involved in regulating nitrogen metabolism; however it is believed that it does so through inhibition of translation rather than impacting mRNA stability [81]. Another RNA binding protein, circadian controlled translational regulator (CCTR), has been identified in the dinoflagellate *Lingulodinium polyedrum *and likewise regulates translation of luciferin binding protein [82]. Increasingly, RNA binding proteins are recognized to play a role in mRNA stability in many systems, which has significant impacts on measurements of gene expression [83]. A recent study in the toxic dinoflagellate *Alexandrium catenella *identified several RNA binding proteins proposed to be involved in RNA silencing and other post-transcriptional regulation mechanisms [84]. The up-regulation of RNA binding proteins at 1 and 4 hours post-P-addition may similarly reflect a role in differential mRNA stability in *K. brevis*. It is also apparent that local changes in chromatin condensation can rapidly alter the availability of genes [41] and thereby modulate levels of transcription [85] even in the apparent absence of regulation by basal transcription factors in dinoflagellates.

## Conclusions

The precedence for post-transcriptional control in dinoflagellates, the absence of canonical transcription regulators, and the presence of the SL on diverse dinoflagellate transcripts suggests that dinoflagellate gene expression is regulated post-transcriptionally. Nonetheless, transcriptome analysis in *K. brevis *was informative of its physiological responses to nutrient depletion and nutrient addition. Of particular interest was the rapid increase in numerous PPR protein transcripts following nutrient addition. The response of these nuclear-encoded regulators of organellar RNA preceded that of the photosystem, suggesting a role in the reactivation of energy production in response to both N- and P-addition. The presence of the SL on PPR protein transcripts further suggests that this "reawakening" is achieved at the post-transcriptional level. Clarification of the contributions of differential rates of *trans*-splicing, differential mRNA stability, and control of chromatin accessibility for transcription is central to understanding dinoflagellate gene expression.

## Competing interests

The authors declare that they have no competing interests.

## Authors' contributions

JSM participated in the design of the study and sample collection, carried out all RNA extractions, array and qPCR analyses, and drafted the manuscript. EAM established the low nutrient adapted cultures. ALK carried out the qPCR assays, MB processed the microarrays, JGJ carried out the growth curve microarray study, and GLH participated in the design of the study and sample collection. FMVD conceived of the study, participated in the design of the study and sample collection, data analysis, and writing the manuscript. All authors read and approved the final manuscript.

## Supplementary Material

Additional file 1 **Primers used for real-time PCR**. This pdf file contains the contig number, sequence description, forward and reverse primer sequences, and annealing temperatures of all genes validated by qPCR in this study. Click here for file

Additional file 2 **Annotated genes in the nitrogen addition trend set**. This pdf file contains the contig number, sequence description, BLASTx e-value, cluster number, and fold change and p-values for all annotated genes in the nitrogen addition trend set. Click here for file

Additional file 3 **Genes from the N-addition trend set in the enriched GO terms involved in amino acid biosynthesis/metabolism, sulfate assimilation and reduction, or nitrogen compound biosynthetic processes**. This pdf file contains the contig number, sequence description, BLASTx e-value, and fold change and p-values for the genes in the N-addition trend set resulting in enrichment of GO terms involved in amino acid biosynthesis/metabolism, sulfate assimilation and reduction, or nitrogen compound biosynthetic processes. Click here for file

Additional file 4 **Annotated genes in the phosphorus addition trend set**. This pdf file contains the contig number, sequence description, BLASTx e-value, cluster number, and fold change and p-values for all annotated genes in the phosphorus addition trend set. Click here for file
