# Supplementary material for: Transcriptomic response of the red tide dinoflagellate, Karenia brevis, to nitrogen and phosphorus depletion and addition
Source: BMC Genomics. 2011 Jul 5;12:346. doi: 10.1186/1471-2164-12-346 (PMC3149589; doi:10.1186/1471-2164-12-346)
Supplement: Additional file 1 — Primers used for real-time PCR. This pdf file contains the contig number, sequence description, forward and reverse primer sequences, and annealing temperatures of all genes validated by qPCR in this study. [file 1471-2164-12-346-S1.PDF]

Additional File 1. Primers used for real-tme PCR.

Sequence Description	Contig #	Forward Primer	Reverse Primer	Annealing Temp	Study
ubiquitin	Contig_117	GGAGCTTTCCGCAGAAATGAGT	CGCAGACACACTTACTTGGCAA	55 °C	N
hypothetical protein	Contig_2004	GAGCAAGCGACGCCAAGATTA	CGACAGGAAGCGAGTTGGAATT	58 °C	P
photosystem ii reaction center protein d1	Contig_2180	CAACGGGGGAACCTACCAAATG	GCTACGCATATCCAAGGACGCA	58 °C	N & P
beta-mannosidase	Contig_3120	CACTTTGGTCAATCGGCATT	GTGGCAGGCAACGTGATTGT	60 °C	P
pentatricopeptiderepeat-containing protein	Contig_3257	GGCTTGTCGCCTGACATATT	TGAGCACCTCACTGAGCAAC	60 °C	N & P
pentatricopeptiderepeat-containing protein	Contig_3556	GTCCTTCTTTCTCGCAGGCT	CAAGCAATGGGAGCGGGCAT	58 °C	P
pentatricopeptiderepeat-containing protein	Contig_3574	CGCCGAGACTGAACTGTTACAA	ACCCGATTGACGCAACTCTT	60 °C	N
triose-phosphate isomerase	Contig_3893	GGAGGGCAACGCACTGAAA	CGCGGGCTGTATGAGACCTT	55 °C	N
replication protein70kda	Contig_4170	CGCCAGCATTGACGCCATA	GGTGCTGTGAAGCTGGTGATGA	58 °C	N
cdc2-like protein kinase	Contig_5041	GGCAGAAGCGTTCTTGGTCTTT	GCTTCAAGTCACGGTGCATGAT	60 °C	N
f-box protein isoform cra_a	Contig_6057	CACTGATCGGAGCCTGCAAA	GACTTGGCGCCCAGAGAGAT	62°C	P
photosystem ii 44 kda protein	Contig_9479	GGTGGTCTGGAAATGCTCGATT	GGTCAACACCCCAGCCTAATGT	58 °C	N
photosystem ii 47 kda protein	Contig_9488	CAGCTGCAACGCCCATTGA	CCCATGCGTCCGAATCTGTT	55°C	P
photosystem ii d2 protein	Contig_10035	CGCCTATTTAGCCGTTGGTGGT	GAAACGGCAGCCGTAAGGAAGT	62°C	P
replication factor c (activator 1) 38kda	Contig_11376	TAGGAAACAAGGACCGGGCAGT	CGCCAGCATCCTCAATGACAAC	58 °C	N
60S ribosomal protein L34-a	Contig_11420	GCACGAGGATTCCAAGACGT	CGCTTCTTCAGCCGCCTGTA	58 °C	P
ATP synthase CF0 A subunit	Contig_11815	CCGTGTACTTCTGGGAATAA	CGGACTTTCTTCTACAATGT	60 °C	N
