# Supplementary material for: Transcriptomic response of the red tide dinoflagellate, Karenia brevis, to nitrogen and phosphorus depletion and addition
Source: BMC Genomics. 2011 Jul 5;12:346. doi: 10.1186/1471-2164-12-346 (PMC3149589; doi:10.1186/1471-2164-12-346)
Supplement: Additional file 2 — Annotated genes in the nitrogen addition trend set. This pdf file contains the contig number, sequence description, BLASTx e-value, cluster number, and fold change and p-values for all annotated genes in the nitrogen addition trend set. [file 1471-2164-12-346-S2.PDF]

Additional File 2. Annotated genes in the nitrogen addition trend set.

Cluster	Contig	Sequence Description	e-value[a]	4 hr FC	4 hr P-value	12 hr FC	12 hr P-value	24 hr FC	24 hr P-value	48 hr FC	48 hr P-value	Unique[b]
1	278	ser thr protein phosphatase family	1.00E-11	1.3283	3.00E-04	1.0964	3.62E-01	-1.2077	4.08E-02	-1.801	6.67E-16	Y
1	492	SnoK-like protein	2.00E-31	1.2338	2.29E-02	1.2878	1.70E-03	-1.1758	4.40E-02	-1.807	3.48E-09	Y
1	854	serine arginine repetitive matrix 1	1.00E-11	1.1482	2.51E-01	-1.1252	3.01E-01	-1.4032	1.30E-03	-1.7189	1.72E-07	Y
1	1755	glyceraldehyde-3-phosphate dehydrogenas	1.00E-158	-1.0625	5.76E-01	-1.0027	9.80E-01	-1.3322	3.60E-03	-1.864	2.06E-05	Y
1	1995	oxidoreductase, aldo/keto reductase family	5.00E-13	1.0779	6.50E-01	1.1954	2.47E-01	-1.2966	6.52E-02	-1.8608	1.72E-05	Y
1	2157	cu zn superoxide dismutase	1.00E-21	1.4708	1.47E-05	1.7096	4.04E-12	1.3821	8.14E-06	-1.2498	7.46E-05	Y
1	2615	octopine dehydrogenase	1.00E-19	-1.1792	5.38E-02	-1.0778	5.61E-01	-1.3799	9.74E-05	-1.8945	4.70E-05	Y
1	3385	DnaJ and TPR domain protein	3.00E-22	-1.056	5.63E-01	1.1247	1.87E-01	1.109	2.24E-01	-1.7888	3.79E-12	Y
1	4372	tetrahydrofolate dehydrogenase/cyclohydr	3.00E-27	-1.0465	6.11E-01	1.066	4.96E-01	-1.1885	5.71E-02	-1.8931	6.08E-06	Y
1	4859	cation channel family protein	1.00E-09	-1.2779	1.45E-02	1.1271	3.06E-01	-1.0775	3.17E-01	-1.7298	6.00E-05	Y
1	4886	m protein	1.00E-04	-1.7151	3.74E-14	-1.0277	9.08E-01	1.012	9.25E-01	-1.0968	6.67E-01	Y
1	5009	calmodulin-like	9.00E-11	-1.2172	4.34E-02	-1.0557	6.65E-01	-1.2396	6.77E-02	-1.7427	4.82E-05	Y
1	5629	predicted protein	2.00E-22	-1.0383	7.50E-01	1.2402	2.50E-02	1.032	7.48E-01	-1.7795	5.01E-13	Y
1	5969	nad-binding rossmann fold oxidoreductase	1.00E-20	1.038	6.61E-01	1.0598	5.68E-01	-1.1046	2.53E-01	-1.8175	5.97E-11	Y
1	6361	rRNA processing protein Pwp1, putative	4.00E-24	1.0527	7.08E-01	-1.0024	9.85E-01	-1.4273	1.56E-02	-1.8387	4.64E-06	Y
1	6490	peptidylglycine alpha-hydroxylating monc	1.00E-12	1.0909	3.61E-01	1.5623	1.49E-06	1.1001	2.55E-01	-1.8175	1.91E-12	Y
1	6912	ADP-ribosylglycohydrolase	2.00E-13	1.2145	7.82E-02	1.2665	2.06E-02	-1.1926	7.94E-02	-1.8645	1.32E-07	Y
1	7071	Integral membrane protein TerC	3.00E-18	1.1264	9.08E-02	1.0767	5.21E-01	1.0695	4.05E-01	-1.9258	1.85E-19	Y
1	7305	Non-ribosomal peptide synthetase module	2.00E-06	-1.0704	5.22E-01	-1.0509	6.78E-01	-1.0205	8.38E-01	-1.9212	3.87E-10	N
1	7587	dna-directed rna polymerase beta subunit 1	1.00E-04	-1.3106	4.49E-02	1.1168	6.47E-01	-1.2517	5.78E-02	-1.7216	1.94E-08	Y
1	7818	ribosome assembly protein 4	1.00E-24	-1.1465	3.41E-01	1.2615	1.95E-02	-1.1706	7.80E-02	-1.7288	7.45E-08	Y
1	7919	serine threonine protein phosphatase	1.00E-08	-1.397	4.34E-02	1.1165	4.25E-01	-1.2316	5.01E-02	-1.7272	1.98E-06	Y
1	8223	hypothetical protein	1.00E-04	-1.5404	7.53E-08	-1.1003	5.32E-02	-1.166	8.00E-04	-1.8541	4.32E-13	Y
1	8347	TPR repeat-containing protein	1.00E-10	1.0609	4.50E-01	1.039	6.27E-01	-1.4148	8.00E-04	-1.9554	5.17E-11	Y
1	8890	lipooligosaccharide cholinephosphotransfe	1.00E-05	1.0954	3.05E-01	1.3415	8.81E-06	1.2873	3.10E-03	-1.7218	5.07E-24	Y
1	8909	aaa family	1.00E-04	-1.7083	3.00E-05	-1.2763	1.32E-01	-1.1891	1.03E-01	-1.2464	3.77E-01	Y
1	9098	sugar o-methyltransferase	1.00E-32	-1.0086	9.57E-01	1.7992	3.21E-06	1.0924	4.17E-01	-1.8248	1.00E-04	Y
1	9122	histone lysine methyltransferase set5	1.00E-16	-1.1328	3.86E-01	1.1474	2.43E-01	1.0552	6.59E-01	-1.8585	2.72E-06	Y
1	9536	chaperone DnaJ domain protein	4.00E-10	-1.2037	5.60E-02	1.1337	2.49E-01	-1.1791	1.21E-02	-1.785	1.56E-07	Y
1	9759	polypeptide n-acetylgalactosaminyltransfe	1.00E-16	1.0184	8.91E-01	1.1166	3.04E-01	1.0565	6.02E-01	-1.8803	3.03E-05	Y
1	10216	monoglyceride lipase	1.00E-08	-1.0246	7.42E-01	-1.015	7.53E-01	-1.1307	1.15E-02	-1.8408	8.46E-17	Y
1	11123	voltage-dependent p q type calcium chann	1.00E-11	-1.1039	9.53E-02	1.0156	8.72E-01	-1.2568	1.12E-07	-1.7677	1.30E-14	Y
1	11538	prolyl 4-hydroxylase alpha subunit2-oxogl	1.00E-04	-1.0241	7.58E-01	1.0875	3.08E-01	-1.2504	2.00E-03	-1.7969	5.64E-19	Y
1	11540	peptidaseacyl-coenzyme a 6-aminopenicill	1.00E-13	-1.0553	7.21E-01	-1.0788	4.95E-01	-1.3999	3.40E-03	-1.8366	1.28E-06	Y
2	191	hypothetical protein	4.00E-09	1.2005	1.86E-02	1.3193	4.60E-03	1.7288	4.88E-13	2.2799	2.87E-22	Y
2	349	had-superfamilysubfamilyvariant 1	1.00E-07	1.2338	3.32E-02	1.268	7.20E-02	1.5741	4.00E-21	1.7233	7.53E-07	Y
2	614	putative amino acid aldolase or racemas…	9.00E-52	1.2374	2.12E-02	1.6024	9.30E-06	2.0718	9.97E-16	1.6866	5.85E-08	Y

2	871	type iii effector protein with ppr repeats	1.00E-11	-1	1.00E+00	1.0811	4.95E-01	1.2899	2.94E-02	1.877	8.79E-07	Y
2	1193	wd-repeat protein	1.00E-19	1.2959	1.80E-03	1.595	1.23E-11	1.4482	9.37E-06	1.744	4.44E-40	Y
2	1460	endonuclease exonuclease phosphatase	1.00E-11	1.0896	3.01E-01	1.3762	1.90E-03	1.5262	1.24E-06	2.1506	1.76E-16	Y
2	1602	acylneuraminate cytidylyltransferase	4.00E-53	1.2537	5.87E-02	1.2423	2.47E-02	1.5909	1.01E-05	1.8958	2.60E-08	Y
2	1919	60s ribosomal protein l7	1.00E-40	1.0053	9.66E-01	1.1136	3.78E-01	1.6795	2.58E-05	2.5073	2.50E-11	Y
2	2133	aaa atpase domain-containing protein	1.00E-11	1.433	9.54E-05	1.778	1.55E-08	1.356	4.00E-04	1.3238	1.14E-05	Y
2	2272	hypothetical protein	1.00E-04	1.469	1.31E-01	1.3372	1.36E-01	2.6157	2.00E-05	5.4715	2.12E-10	N
2	2627	Large exoproteins involved in h...	2.00E-06	1.4245	3.46E-05	1.6913	2.00E-06	1.9408	2.62E-12	1.8132	3.55E-11	Y
2	2800	wd-repeat protein	1.00E-06	1.3741	1.87E-07	1.5768	1.98E-05	1.6791	2.49E-14	1.7275	2.47E-25	Y
2	2853	protein with PPR repeats	2.00E-22	1.253	4.79E-02	2.1457	1.09E-05	1.2816	7.49E-02	1.3473	1.44E-01	N
2	3079	lung alpha beta hydrolase protein 1	1.00E-09	1.0606	5.64E-01	1.5084	3.48E-02	1.9003	8.51E-11	1.2303	7.88E-02	Y
2	3234	pentatricopeptiderepeat-containing protein	1.00E-10	1.0479	6.17E-01	1.0525	3.79E-01	1.5855	9.96E-15	2.0482	1.42E-12	Y
2	3267	putative type III effector protein with ppr..	9.00E-09	1.8347	2.03E-05	1.9201	2.91E-10	1.3081	4.40E-03	1.3088	1.42E-02	Y
2	3277	pentatricopeptiderepeat-containing protein	1.00E-24	1.5538	7.40E-03	1.9734	2.62E-05	1.3201	2.08E-02	1.2738	1.91E-02	Y
2	3789	60s acidic ribosomal protein p0	1.00E-17	1.1117	6.03E-01	1.0958	6.46E-01	1.346	7.74E-02	2.1434	1.41E-05	N
2	4138	homocysteine s-methyltransferase	1.00E-18	1.3315	5.25E-07	1.5934	2.00E-04	1.6871	7.85E-11	1.8642	1.75E-19	Y
2	4221	prolyl 4-hydroxylase	6.00E-11	1.4962	5.71E-06	1.7799	3.97E-11	1.3696	1.77E-05	1.2252	1.12E-02	Y
2	4242	leucine rich repeat family protein	1.00E-04	1.0362	6.61E-01	1.4438	3.28E-05	1.5574	5.15E-08	1.7624	1.64E-06	Y
2	4416	isochorismatase hydrolase	1.00E-52	1.1996	2.89E-02	1.2668	2.05E-02	1.7777	2.76E-15	1.2312	1.88E-02	Y
2	4573	mitochondrial carrier	1.00E-04	1.7051	4.29E-14	1.031	7.11E-01	1.3861	8.82E-14	1.5946	1.32E-07	Y
2	4833	ferulic acid esterase	1.00E-08	1.0507	6.18E-01	-1.0292	8.36E-01	1.284	3.94E-02	1.7056	6.11E-06	Y
2	4851	nucleoporin 107	1.00E-04	-1.0578	6.82E-01	1.338	6.21E-02	1.4637	9.17E-07	1.7788	1.10E-08	Y
2	4928	pentatricopeptiderepeat-containing protein	1.00E-18	-1.2067	1.90E-01	1.5571	8.87E-02	1.7274	8.21E-05	2.0208	1.03E-02	N
2	5153	chromosome segregation protein smc	1.00E-08	-1.0521	6.28E-01	1.6308	1.40E-02	1.8327	2.19E-06	1.4243	1.07E-02	Y
2	5229	hypothetical protein Tb10.6k15.3460	4.00E-78	-1.1128	4.13E-01	-1.0732	7.22E-01	1.2907	9.77E-02	1.8996	1.66E-07	Y
2	5415	osm3-like kinesin	1.00E-10	1.1165	4.13E-01	1.7544	2.49E-06	1.5827	2.00E-04	1.603	4.46E-05	Y
2	5432	tpa_inf: hdc02849	1.00E-06	1.1047	3.43E-01	1.5433	2.16E-06	2.0307	4.49E-14	2.2149	6.33E-08	Y
2	5496	tb-292 membrane associated protein	1.00E-08	1.2044	4.64E-01	-1.3666	1.76E-01	2.4086	1.51E-02	6.5189	1.02E-07	Y
2	5703	acetyltransferase-like protein	1.00E-11	1.0214	7.76E-01	1.1108	1.63E-01	1.4515	1.99E-07	1.8283	4.01E-11	Y
2	6049	kinesin-like calmodulin-binding protein	1.00E-40	1.278	1.55E-01	1.6679	6.90E-03	1.7766	5.87E-05	2.2211	3.65E-10	N
2	6110	ankyrin domain protein ank2	1.00E-14	1.5227	1.20E-03	1.436	8.35E-02	1.839	1.59E-06	1.5267	2.11E-02	Y
2	6135	novel proteincatechol-o-methyltransferase	1.00E-39	1.3018	1.01E-06	1.7603	8.90E-34	1.9028	6.04E-23	1.894	5.76E-20	Y
2	6508	ribosomal large subunit pseudouridine syn	1.00E-25	1.1558	2.56E-01	1.4352	1.47E-02	1.6404	9.13E-05	1.7997	8.68E-09	Y
2	6569	Serine-threonine protein kinase that is pa..	9.00E-17	1.2413	6.68E-02	1.28	2.02E-02	1.5078	1.00E-04	1.8655	9.86E-07	Y
2	6655	chromosome segregation protein smc	1.00E-06	-1.0096	9.31E-01	1.7728	1.73E-05	2.0991	2.16E-07	1.3366	7.30E-03	Y
2	6658	hypothetical membrane spanning protein	1.00E-05	1.0982	5.13E-01	1.1598	2.14E-01	1.3104	2.43E-02	1.7349	4.72E-05	Y
2	6693	s-adenosyl-methyltransferase	1.00E-25	-1.0674	4.49E-01	1.1731	2.58E-01	1.4621	4.00E-04	1.7535	2.76E-06	Y
2	6748	vanin 1	1.00E-32	1.1397	2.48E-01	1.2689	2.91E-02	1.5643	9.93E-05	1.9185	5.68E-09	Y
2	6752	30s ribosomal protein s1	1.00E-23	1.087	3.57E-01	-1.7215	2.28E-06	1.2251	4.96E-02	1.5959	3.15E-10	Y
2	6783	monooxygenase family protein	1.00E-06	1.0675	4.02E-01	1.1727	4.60E-03	1.4097	9.74E-11	1.8333	4.70E-15	Y

2	7133	adp-ribosyl-(dinitrogen reductase) hydrola	1.00E-12	-1.0052	9.49E-01	1.2608	5.90E-03	1.7322	7.34E-10	1.0462	7.48E-01	Y
2	7206	calcium-dependent protein kinase	1.00E-40	1.0947	4.51E-01	1.1098	3.92E-01	1.3977	1.20E-03	1.8711	8.85E-07	Y
2	7284	riken cdna 5630401d24	1.00E-09	1.7013	1.14E-06	1.1922	1.13E-01	1.157	6.89E-02	1.4117	4.90E-03	Y
2	7362	lipoprotein, putative	3.00E-25	-1.003	9.91E-01	1.8345	1.81E-05	1.2073	3.05E-01	1.696	3.00E-04	Y
2	7416	fad dependent oxidoreductase	1.00E-06	-1.1214	4.26E-01	1.702	4.82E-05	1.6758	2.33E-28	1.4092	2.49E-02	Y
2	7478	cystathionine beta-synthase	1.00E-58	1.2072	3.03E-02	1.2094	1.14E-02	1.396	6.07E-06	1.8039	2.80E-14	Y
2	7571	17-beta hydroxysteroid dehydrogenase typ	3.00E-12	1.1733	1.36E-02	1.107	2.63E-01	1.4838	7.46E-10	2.0347	6.49E-23	Y
2	7702	possible esterase lipoprotein lpqc	1.00E-11	1.1339	7.62E-02	1.2014	3.64E-02	1.6471	6.43E-09	1.8564	4.79E-11	N
2	7803	calcium-dependent protein kinase	1.00E-27	1.1757	5.65E-02	1.1489	1.14E-01	1.5414	7.52E-07	1.7507	1.27E-19	Y
2	7918	hypothetical protein	1.00E-13	1.2745	1.46E-02	1.6112	2.68E-07	1.8017	9.13E-11	1.4679	4.00E-04	Y
2	8381	glucose-repressible alcohol dehydrogenase	1.00E-12	1.2613	7.10E-03	1.4468	8.48E-06	1.9353	1.41E-14	2.0348	9.46E-14	Y
2	8382	glucose-repressible alcohol dehydrogenase	1.00E-14	1.1495	2.51E-01	1.5161	2.00E-04	2.0615	2.58E-07	2.0841	1.69E-14	N
2	8803	formamidopyrimidine-dna glycosylase	1.00E-29	-1.4334	8.35E-02	-1.1022	5.77E-01	1.8994	3.00E-04	2.7781	2.47E-09	Y
2	8834	predicted protein	8.00E-05	1.5515	1.98E-05	1.9768	3.25E-12	1.3117	1.20E-03	1.1651	2.26E-01	Y
2	8932	photosystem i p700 chlorophyll a apoprote	1.00E-66	1.0914	7.47E-01	1.0444	8.16E-01	1.7875	1.30E-03	4.0065	3.21E-05	N
2	9019	astacin-like protein	1.00E-14	1.2711	2.04E-02	1.7132	6.85E-07	1.4049	7.00E-04	1.3256	8.80E-03	Y
2	9038	Vegetative cell wall protein gp1precur...	1.00E-05	1.1334	1.46E-01	1.6076	2.43E-08	1.7371	1.81E-10	1.2006	2.43E-02	Y
2	9132	hypothetical protein	4.00E-08	1.102	4.51E-01	1.4502	2.00E-04	1.8937	1.59E-33	1.2753	8.00E-04	Y
2	9275	unnamed protein product	2.00E-06	2.0386	2.37E-24	1.053	6.50E-01	1.4587	6.53E-08	1.4681	5.90E-03	Y
2	9438	glutathionylspermidine synthase	1.00E-37	-1.2707	5.64E-02	1.2396	2.34E-01	1.7306	7.32E-07	1.3227	1.98E-01	Y
2	9479	photosystem ii 44 kda protein	1.00E-170	1.0577	7.42E-01	-1.1817	4.79E-01	-1.0414	8.05E-01	2.3597	9.62E-05	N
2	9599	methyltransferase protein	1.00E-17	-1.1569	3.55E-01	1.6498	2.80E-03	1.9303	5.80E-08	1.1837	3.07E-01	Y
2	9606	abc-type multidrug transporter with fused	1.00E-21	-1.1785	6.99E-02	-1.0167	8.39E-01	1.4623	2.59E-07	1.9898	2.45E-27	Y
2	9651	serine-type d-ala-d-ala carboxypeptidase	1.00E-09	1.3168	3.13E-02	1.5348	3.10E-03	2.0739	1.94E-12	2.3764	4.70E-13	Y
2	9784	5-azacytidine resistance	1.00E-10	1.0876	3.42E-01	-1.0596	6.24E-01	1.364	7.00E-04	1.7003	2.37E-09	Y
2	10042	hypothetical protein	1.00E-06	-1.0766	4.82E-01	1.2809	9.00E-03	1.9015	1.15E-16	2.0116	2.28E-06	Y
2	10146	60s ribosomal protein l21	1.00E-66	1.1637	2.86E-01	1.2442	8.01E-02	1.6253	1.00E-04	2.6964	7.81E-08	N
2	10248	dual specificity phosphatase 2	1.00E-11	1.8138	9.25E-08	1.7806	2.83E-07	1.276	1.26E-02	1.4115	1.20E-03	Y
2	10425	formamidopyrimidine-dna glycosylase	1.00E-27	-1.1275	4.50E-03	-1.1278	3.34E-02	1.4608	1.31E-15	1.7056	2.18E-16	Y
2	10631	Probable DNA gyrase subunit A, chlorop..	7.00E-43	1.7448	6.60E-10	1.4618	5.70E-03	1.0748	5.12E-01	1.0646	6.51E-01	Y
2	10668	wd-repeat protein	1.00E-13	1.0976	4.85E-01	1.0895	4.96E-01	1.3744	9.80E-03	1.9627	1.35E-07	Y
2	10917	AhpC/TSA family protein	4.00E-10	1.2277	7.80E-03	-1.1035	4.10E-01	1.2552	1.30E-03	2.0132	7.44E-11	Y
2	10919	cellulose binding elicitor lectin	1.00E-05	1.1958	5.07E-02	1.5853	7.67E-06	1.9204	1.20E-10	1.8604	6.06E-05	Y
2	10930	hydrolase TatD family protein	2.00E-18	1.1265	2.75E-01	1.1781	9.95E-02	1.5385	8.88E-07	1.9931	3.54E-12	Y
2	11073	endo-1,4-beta-xylanase D precursor	1.00E-23	1.2055	2.42E-01	1.352	6.03E-02	1.9077	2.16E-06	1.6323	4.27E-05	Y
2	11151	predicted protein	9.00E-07	1.4862	2.93E-05	1.4908	1.43E-05	1.7627	9.34E-10	2.3284	1.73E-14	Y
2	11198	tpr repeat protein	1.00E-05	-1.3095	1.58E-01	1.2928	1.38E-01	1.7697	2.42E-05	1.609	2.77E-02	Y
2	11237	60S ribosomal L22e protein, putative	4.00E-16	1.1632	3.75E-01	1.0994	5.31E-01	1.5723	6.80E-03	2.7677	3.03E-09	Y
2	11417	dead (asp-glu-ala-asp) box polypeptide 51	1.00E-12	-1.2886	2.86E-02	1.6159	9.00E-03	1.8343	6.65E-14	1.2892	1.04E-01	N
2	11468	2h2 type family protein	1.00E-05	-1.0018	9.86E-01	1.4896	4.00E-04	1.7519	2.99E-08	1.4334	9.00E-04	Y

2	11682	2og-feoxygenase family protein	1.00E-15	1.1161	2.68E-01	1.2782	3.42E-02	1.3602	2.00E-04	1.719	9.07E-06	Y
2	11775	HEAT repeat	6.00E-06	1.2478	7.18E-02	1.5783	8.19E-07	1.5391	1.89E-07	1.7878	1.20E-09	Y
2	11815	atp synthase cf0 a subunit	1.00E-11	1.0541	8.32E-01	-1.1135	6.47E-01	2.5999	7.89E-13	5.9295	8.34E-05	N
2	11850	photosystem i p700 chlorophyll a apoprote	1.00E-105	1.1349	4.99E-01	-1.0137	9.27E-01	1.4974	2.90E-03	3.9851	2.90E-08	N
3	114	atp synthase f0 subunit c	1.00E-12	1.1676	2.11E-01	-1.2627	1.60E-01	-1.4433	1.17E-02	-1.8531	6.03E-09	Y
3	642	hypothetical protein	1.00E-21	-1.0717	4.58E-01	-1.244	3.03E-02	-1.8438	1.26E-08	-2.2729	3.21E-14	N
3	765	ORF137	6.00E-16	-1.0833	7.06E-01	-1.3244	1.85E-01	-3.0841	1.87E-08	-2.9744	4.70E-03	Y
3	1654	chloroplast light harvesting protein isoforr	1.00E-74	-1.2845	8.20E-03	-1.28	6.86E-06	-1.7958	1.19E-06	-2.8986	0.00E+00	N
3	1797	cation channel family protein	1.00E-05	-1.1434	3.65E-01	-1.454	1.20E-03	-1.7322	3.30E-09	-1.2131	2.52E-01	Y
3	1879	ATP synthase F0 subunit C	1.00E-13	1.1125	3.25E-01	-1.3153	1.96E-02	-1.4237	8.00E-04	-1.8052	3.43E-07	Y
3	2164	plastid ferredoxin nadph reductase protein	1.00E-71	-1.0126	8.76E-01	-1.1964	3.68E-02	-1.9169	8.65E-12	-2.2217	2.64E-07	Y
3	2519	FtsJ homolog 3	4.00E-59	-1.1663	6.71E-02	-1.6748	3.62E-14	-2.255	5.64E-12	-1.7396	7.94E-09	Y
3	2796	hypothetical protein	9.00E-39	-1.0674	6.18E-01	-1.2494	1.59E-02	-1.7781	1.08E-10	-2.3701	1.37E-11	Y
3	2845	immunity-related gtpasecinema 1	1.00E-09	-1.3229	2.80E-02	-1.3519	5.00E-04	-1.6669	3.02E-06	-2.3165	1.89E-18	Y
3	2916	UDP-glucose dehydrogenase, putative	1.00E-125	-1.0167	8.96E-01	-1.223	1.85E-01	-1.6289	5.22E-05	-2.2811	5.55E-14	Y
3	2918	udp-glucose dehydrogenase	1.00E-38	1.0166	8.85E-01	-1.2177	2.25E-01	-1.6889	1.82E-05	-2.1881	8.39E-11	Y
3	2933	prli-interacting factor l-like	1.00E-92	-1.1605	3.58E-02	-1.0894	3.82E-01	-1.5172	2.00E-04	-1.948	7.44E-11	Y
3	3363	hypothetical protein	9.00E-08	1.0233	8.56E-01	-1.4165	1.83E-02	-1.5541	1.20E-03	-1.9747	4.17E-07	Y
3	3574	pentatricopeptiderepeat-containing protein	1.00E-19	1.4211	4.29E-06	-2.1658	3.88E-17	-1.671	4.56E-17	-1.2957	1.40E-03	Y
3	3653	ribosomal protein s2	1.00E-15	1.0398	8.35E-01	-1.0876	7.87E-01	-1.7522	4.74E-07	-1.2796	2.68E-01	N
3	3747	cobalamin synthesisp47k	1.00E-78	-1.2938	3.60E-02	-1.4293	2.00E-04	-2.0813	2.39E-27	-1.837	2.81E-11	Y
3	3893	triose-phosphate isomerase	1.00E-103	1.0066	9.30E-01	-1.6387	5.00E-04	-2.4218	5.50E-11	-3.9681	1.63E-11	Y
3	4561	oligosaccharyl transferase-like protein	1.00E-25	-1.0967	6.41E-01	-1.1592	4.22E-01	-1.567	9.40E-03	-2.1774	2.01E-05	Y
3	4809	plastid soluble inorganic pyrophosphatase	1.00E-103	1.0628	3.71E-01	-1.2018	4.43E-02	-1.7834	3.80E-13	-2.5638	1.22E-17	Y
3	5264	thioredoxin domain-containing protein 5 p	1.00E-46	-1.0235	8.64E-01	-1.7326	3.64E-06	-1.7533	1.61E-06	-1.9003	4.85E-07	N
3	5340	cgmp-dependent protein kinase	1.00E-19	-1.3527	2.81E-02	-1.2395	3.00E-01	-1.4896	2.20E-03	-1.7075	2.21E-05	Y
3	5584	succinate-ligase (adp-forming)	1.00E-95	-1.1075	3.66E-01	-1.4078	1.07E-01	-1.4501	2.96E-02	-1.8729	2.39E-05	Y
3	5604	3-phosphoadenosine-5-phosphosulfate red	1.00E-43	-1.106	4.78E-01	-1.1878	2.16E-01	-1.7132	3.00E-04	-2.2413	8.24E-07	Y
3	5605	3-phosphoadenosine-5-phosphosulfate red	1.00E-36	-1.0617	7.24E-01	-1.527	1.18E-02	-2.6187	1.36E-06	-3.3197	3.81E-06	N
3	5773	dual specificity protein phosphatase family	6.00E-10	1.0954	4.01E-01	-1.0997	3.81E-01	-1.5194	5.00E-04	-1.7808	4.19E-07	Y
3	6069	ornithine carbamoyltransferase family p...	1.00E-57	-2.0479	4.00E-27	-1.3705	1.08E-06	-1.3123	4.00E-04	-1.1902	1.62E-01	Y
3	6089	MPBQ/MSBQ transferase cyanobacterial t	8.00E-07	-1.0673	6.24E-01	-2.7252	6.37E-14	-2.2666	1.21E-09	-1.5343	3.90E-03	N
3	6121	tubulin-tyrosine ligase family protein	1.00E-25	-1.1777	2.83E-01	-1.7979	1.14E-07	-1.34	1.23E-01	-1.1146	3.03E-01	Y
3	6452	pre-rrna processing protein	1.00E-09	-1.4159	3.64E-02	-1.1278	5.40E-01	-1.7501	6.00E-04	-2.2439	7.23E-06	Y
3	6523	tpr repeat-containing protein	1.00E-05	-1.1087	6.19E-01	-1.1031	4.86E-01	-1.957	4.70E-08	-2.6967	8.52E-07	N
3	6531	methyltransferase type 11	1.00E-28	-1.0501	7.77E-01	-2.0414	7.48E-13	-2.0257	7.09E-08	-1.1392	4.29E-01	N
3	6970	homoserine dehydrogenase	1.00E-41	-1.3951	1.60E-03	-1.349	3.88E-05	-1.4863	7.04E-05	-2.0356	7.36E-05	Y
3	7353	suppressor of ascus dominance	1.00E-08	-1.1845	3.00E-01	-1.4455	1.50E-02	-1.7696	1.00E-04	-2.1678	2.26E-06	Y
3	7364	unc-13 homolog b	1.00E-10	-1.6651	4.60E-03	-1.7297	3.69E-02	-1.7939	2.54E-02	-1.7537	3.68E-09	Y
3	7593	recombination activating protein 1	1.00E-11	-1.1813	2.68E-01	-1.2249	1.23E-01	-1.7546	2.89E-05	-1.6617	6.00E-04	Y

3	7603	cgmp-dependent protein kinase	1.00E-43	-1.1071	2.83E-01	-1.2326	3.90E-02	-1.4365	4.42E-05	-1.7462	3.32E-08	Y
3	7738	H+-translocating inorganic pyrophosphata	9.00E-40	-1.1235	3.30E-01	-1.1381	2.40E-01	-1.4746	1.20E-03	-2.1592	2.34E-05	Y
3	7776	phosphoglucomutase	2.00E-42	-1.1994	9.76E-02	-1.3978	3.40E-03	-1.6395	2.00E-04	-1.7804	6.93E-08	Y
3	7916	dna repair protein rad50	1.00E-65	-1.4362	1.22E-02	-1.7575	3.00E-04	-1.8915	4.35E-05	-1.6217	9.55E-06	Y
3	8119	ribosomal rna processing 12 homolog	1.00E-05	-1.1219	1.64E-02	1.0248	8.25E-01	-1.639	5.61E-07	-1.983	1.46E-31	Y
3	8548	DEAD/DEAH box helicase and helicase c	3.00E-28	-1.3403	9.07E-05	-1.1822	5.71E-02	-1.4037	1.10E-03	-1.7041	1.82E-07	Y
3	8900	zinc finger ccch-type containing 10	1.00E-04	-1.0523	6.11E-01	-1.0144	8.57E-01	-1.7199	3.26E-10	-1.5311	1.20E-03	Y
3	9101	severin kinase	7.00E-23	-1.1601	2.87E-01	-1.1	4.00E-01	-1.5336	1.39E-02	-2.0599	1.11E-05	Y
3	9157	ribonuclease h	1.00E-25	-1.1818	9.07E-02	-1.4728	4.59E-05	-1.8771	4.69E-13	-1.3567	1.15E-06	Y
3	9282	isoform cra_a	1.00E-12	-1.7511	3.13E-06	-1.3905	1.36E-01	-1.3515	9.31E-02	-1.4872	1.11E-02	Y
3	9847	gamma-glutamyl hydrolase (conjugase, fo	2.00E-49	1.1085	3.04E-01	-1.5096	5.00E-04	-1.7412	5.59E-07	-1.2693	3.16E-02	Y
3	9851	ribosomal protein L16	1.00E-31	1.1305	5.02E-01	-1.016	9.42E-01	-1.8073	1.61E-05	-1.168	3.65E-01	N
3	10258	hypothetical protein TA12215 [Theileria a	2.00E-31	-1.016	8.94E-01	-1.6904	1.20E-09	-1.8183	1.49E-10	-1.8463	6.69E-06	Y
3	10366	amidases related to nicotinamidase	1.00E-32	-1.1384	1.83E-01	-1.5172	7.77E-06	-1.775	4.71E-12	-1.4414	3.59E-07	Y
3	10479	probable oxidoreductase protein	1.00E-04	-1.2408	1.23E-02	-1.4381	1.38E-06	-1.6565	1.88E-10	-2.0776	2.96E-06	Y
3	10543	super cysteine rich protein; SCRP [Homo	4.00E-10	-1.0243	9.30E-01	-1.6028	9.46E-02	-2.1648	9.69E-08	-1.5535	5.03E-02	N
3	11186	zinc finger protein	1.00E-04	-1.4465	9.19E-02	-1.9571	3.16E-05	-2.1752	5.14E-06	-1.2656	2.06E-01	N
3	11211	WSSV021 [shrimp white spot syndrome v	5.00E-20	-1.2388	2.60E-03	-1.2669	4.24E-02	-1.6436	8.67E-10	-1.7108	6.53E-07	Y
3	11683	sec-independent protein translocase-like p	1.00E-13	1.1641	5.76E-01	-2.3876	4.03E-05	1.1256	5.79E-01	1.798	7.97E-02	N
4	117	ubiquitin [Heterocapsa triquetra]	2.00E-79	-1.4325	2.00E-04	-1.9625	3.21E-02	-2.1724	4.20E-03	-2.6982	7.24E-30	Y
4	1188	protein disulfide isomerase (prolyl 4-hydro	1.00E-05	-3.4051	1.85E-02	-1.302	6.66E-01	-1.5277	3.74E-01	-4.539	8.88E-05	Y
4	3120	beta-mannosidase [Acidobacteria bacteriu	4.00E-11	-1.8845	5.00E-04	-2.7077	5.16E-06	-3.7072	1.28E-17	-1.3845	5.91E-02	N
4	4180	similar to kinetoplast-associated protein ...	1.00E-10	-1.3205	2.86E-01	-2.3529	9.00E-04	-2.7654	1.61E-05	-2.7767	7.60E-09	N
4	6566	calrectulin [Heterocapsa triquetra]	6.00E-30	-2.1912	1.33E-07	-3.0376	3.06E-15	-3.332	1.36E-33	-3.3222	6.41E-36	N
4	9310	cleavage and polyadenylation specificity f	1.00E-63	-1.569	2.50E-01	-3.1853	1.82E-05	-3.0756	6.28E-05	-1.861	5.70E-03	N
5	95	proliferating cell nuclear antigen	1.00E-125	1.3494	2.53E-01	2.5078	1.00E-03	5.727	1.34E-11	10.4812	1.38E-16	N
5	183	pentatrichopeptide repeatprotein	1.00E-06	3.4503	6.36E-31	4.6093	3.97E-23	2.8094	5.62E-28	2.6848	2.42E-12	N
5	467	80 kda protein	1.00E-04	1.7651	2.80E-07	1.9387	4.36E-06	2.0034	1.89E-09	2.2579	3.28E-13	Y
5	597	pentatricopeptiderepeat-containing protein	1.00E-11	1.4653	3.80E-03	2.4661	5.67E-14	1.5969	1.70E-03	1.8849	1.05E-08	Y
5	1745	peptidase d	1.00E-58	1.4232	8.39E-08	1.5199	3.01E-02	2.0653	1.01E-22	1.5834	3.94E-09	Y
5	3061	ENSANGP00000023827 [Anopheles gam	3.00E-10	1.7442	1.00E-04	2.3391	4.65E-10	1.4592	8.00E-04	1.4602	7.00E-04	Y
5	3254	type iii effector protein with ppr repeats	1.00E-15	1.7342	6.39E-14	2.4161	4.17E-16	2.0555	1.47E-19	1.9764	1.97E-11	N
5	3255	pentatricopeptiderepeat-containing protein	1.00E-24	1.3879	1.00E-04	1.9315	1.49E-11	1.7182	3.20E-10	1.7062	1.03E-06	N
5	3257	pentatricopeptiderepeat-containing protein	1.00E-24	1.8692	1.59E-11	2.7847	1.91E-14	2.1042	1.01E-14	1.8699	1.91E-05	N
5	3258	pentatricopeptide (PPR) repeat-containing	2.00E-36	1.6636	4.19E-07	2.4327	1.72E-14	1.958	2.88E-12	1.9379	1.99E-12	N
5	3262	ENSANGP00000029120 [Anopheles gam	1.00E-04	1.5974	2.59E-08	2.1592	3.07E-20	1.5978	6.42E-11	1.5912	7.18E-08	N
5	3266	pentatricopeptide (ISS) [Ostreococcus tau	4.00E-06	1.6862	2.07E-20	2.5536	2.49E-31	2.0946	1.27E-18	2.0616	8.63E-12	N
5	3270	putative type III effector protein with ppr..	3.00E-12	1.7915	2.00E-04	2.1548	2.75E-07	1.4237	6.24E-02	1.7759	2.00E-04	Y
5	3271	pentatricopeptide (PPR) repeat-containing	5.00E-11	1.5236	3.31E-05	2.4592	1.95E-17	2.0907	3.13E-14	2.0751	1.51E-11	N
5	3278	putative type III effector protein with ppr..	1.00E-28	1.6823	1.62E-07	2.486	1.26E-14	1.9206	9.22E-11	1.7414	2.87E-06	N

5	3279	pentatricopeptiderepeat-containing protein	1.00E-29	1.5358	5.00E-04	2.2838	7.62E-09	1.7773	4.51E-05	1.8743	4.08E-06	N
5	3280	pentatricopeptiderepeat-containing protein	1.00E-21	1.9686	1.22E-10	2.7694	1.86E-12	1.6012	2.56E-05	1.6585	6.00E-04	N
5	3281	pentatricopeptiderepeat-containing protein	1.00E-04	1.7386	1.30E-08	2.8109	2.90E-05	1.1941	1.69E-01	1.4165	2.00E-04	N
5	3287	pentatricopeptiderepeat-containing protein	1.00E-21	1.3609	2.16E-08	1.9173	4.00E-23	1.8308	3.99E-30	1.7946	8.83E-13	Y
5	3289	pentatrichopeptide repeatprotein	1.00E-06	2.2851	2.20E-03	3.3332	1.42E-05	1.0687	7.71E-01	1.1176	6.24E-01	N
5	3291	pentatricopeptiderepeat-containing protein	1.00E-33	1.4489	3.53E-07	2.1946	7.12E-19	2.0491	3.59E-19	1.881	1.00E-14	N
5	3292	pentatricopeptiderepeat-containing protein	1.00E-28	1.5703	6.98E-06	2.1678	2.56E-10	1.8906	7.20E-09	1.8567	2.28E-07	N
5	3293	pentatricopeptiderepeat-containing protein	1.00E-17	1.585	1.10E-05	1.9048	3.37E-10	1.8023	6.60E-13	1.5992	4.55E-06	N
5	3295	PUTATIVE PPR REPEATS CONTAININ	5.00E-06	1.8214	2.19E-06	1.9908	9.61E-07	1.2965	3.32E-02	1.3899	5.00E-04	N
5	3307	protein with PPR repeats [Chlamydomona	2.00E-23	1.3486	8.00E-04	2.1893	2.54E-16	1.6908	4.09E-06	1.9384	1.84E-11	N
5	3556	pentatricopeptiderepeat-containing protein	1.00E-25	1.3221	5.60E-03	2.0649	3.29E-09	1.7313	1.55E-09	1.7697	3.57E-10	Y
5	3973	predicted mitochondrial protein [Chlamy..	4.00E-33	1.6237	3.27E-08	2.7309	7.73E-14	1.8869	5.09E-07	1.9281	5.25E-09	N
5	5784	cupin 4 family protein	1.00E-09	1.8835	9.61E-12	1.8989	6.88E-07	1.8572	6.28E-20	1.5988	1.00E-04	Y
5	6547	helicase domain protein	1.00E-16	1.5037	2.08E-02	5.0514	5.99E-12	2.0158	2.90E-03	-1.0478	7.41E-01	Y
5	7156	plastid RNA helicase VDL protein precurs	1.00E-109	2.1783	2.10E-03	5.5036	3.64E-44	1.7363	3.90E-03	1.2947	1.61E-01	Y
5	7526	30s ribosomal protein s4	1.00E-05	2.7994	5.00E-04	2.6917	4.00E-04	3.1252	7.80E-13	4.2543	9.33E-06	N
5	7756	radical s-adenosyl methionine and flavodo	1.00E-43	3.1458	0.00E+00	3.9805	0.00E+00	1.6427	3.48E-19	-1.3046	1.55E-02	Y
5	8569	prolylcarboxypeptidase (angiotensinase c)	1.00E-28	1.3091	1.04E-01	2.0768	5.65E-10	2.3324	2.55E-19	1.9584	1.30E-10	Y
5	9100	plastid glutamate 1-semialdehyde 2,1-amii	1.00E-117	1.0411	6.97E-01	1.8926	5.00E-04	2.7962	4.36E-17	2.6033	3.91E-18	Y
5	9366	hypothetical protein PM8797T_10994 [Pla	2.00E-06	2.8747	6.45E-14	2.6385	1.84E-09	1.6977	3.76E-06	1.4777	8.00E-04	Y
5	11847	GTP-binding protein LepA [Synechocystis	1.00E-25	2.0619	7.11E-08	3.4578	3.03E-10	1.4517	1.33E-02	-1.0378	7.88E-01	Y

[a]e-value of top BLASTx hit

[b]Unique to N-addition trend set

The time points at which a probe qualified for inclusion in the trend set are indicated in red (up) or green (down)

FC: fold change
