# Supplementary material for: Transcriptomic response of the red tide dinoflagellate, Karenia brevis, to nitrogen and phosphorus depletion and addition
Source: BMC Genomics. 2011 Jul 5;12:346. doi: 10.1186/1471-2164-12-346 (PMC3149589; doi:10.1186/1471-2164-12-346)
Supplement: Additional file 3 — Genes from the N-addition trend set in the enriched GO terms involved in amino acid biosynthesis/metabolism, sulfate assimilation and reduction, or nitrogen compound biosynthetic processes. This pdf file contains the contig number, sequence description, BLASTx e-value, and fold change and p-values for the genes in the N-addition trend set resulting in enrichment of GO terms involved in amino acid biosynthesis/metabolism, sulfate assimilation and reduction, or nitrogen compound biosynthetic processes. [file 1471-2164-12-346-S3.PDF]

Additional File 3. Genes from the N-addition trend set in the enriched GO terms involved in amino acid biosynthesis/metabolism, sulfate assimilation and reduction, or nitrogen compound biosynthetic processes.

Contig	Sequence Description	e-value[a]	4 hr FC	4 hr P-value	12 hr FC	12 hr P-value	24 hr FC	24 hr P-value	48 hr FC	48 hr P-value
5605	3-phosphoadenosine-5-phosphosulfate reductase	1.00E-36	-1.0617	7.24E-01	-1.527	1.18E-02	-2.6187	1.36E-06	-3.3197	3.81E-06
5604	3-phosphoadenosine-5-phosphosulfate reductase	1.00E-43	-1.106	4.78E-01	-1.1878	2.16E-01	-1.7132	3.00E-04	-2.2413	8.24E-07
6970	homoserine dehydrogenase	1.00E-41	-1.3951	1.60E-03	-1.349	3.88E-05	-1.4863	7.04E-05	-2.0356	7.36E-05
6069	ornithine carbamoyltransferase family p...	1.00E-57	-2.0479	4.00E-27	-1.3705	1.08E-06	-1.3123	4.00E-04	-1.1902	1.62E-01
7478	cystathionine beta-synthase	1.00E-58	1.2072	3.03E-02	1.2094	1.14E-02	1.396	6.07E-06	1.8039	2.80E-14
4138	homocysteine s-methyltransferase	1.00E-18	1.3315	5.25E-07	1.5934	2.00E-04	1.6871	7.85E-11	1.8642	1.75E-19
6135	novel proteincatechol-o-methyltransferase comt…	1.00E-39	1.3018	1.01E-06	1.7603	8.90E-34	1.9028	6.04E-23	1.894	5.76E-20

[a]e-value of top BLASTx hit

FC: fold change
