# Supplementary material for: Transcriptomic response of the red tide dinoflagellate, Karenia brevis, to nitrogen and phosphorus depletion and addition
Source: BMC Genomics. 2011 Jul 5;12:346. doi: 10.1186/1471-2164-12-346 (PMC3149589; doi:10.1186/1471-2164-12-346)
Supplement: Additional file 4 — Annotated genes in the phosphorus addition trend set. This pdf file contains the contig number, sequence description, BLASTx e-value, cluster number, and fold change and p-values for all annotated genes in the phosphorus addition trend set. [file 1471-2164-12-346-S4.PDF]

Additional File 4. Annotated genes in the phosphorus addition trend set.

Cluster	Contig	Sequence Description	e-value[a]	1 hr FC	1 hr P-value	4 hr FC	4 hr P-value	24 hr FC	24 hr P-value	48 hr FC	48 hr P-value	Unique[b]
1	4180	similar to kinetoplast-associated protein ...	1.00E-10	-1.4382	0.0019	-1.2292	0.2546	-1.1196	0.4011	-1.9427	1.60E-05	N
1	2884	dehydrogenase MVIM-sugar aminotransferase	1.00E-19	-1.3249	0.0835	-1.4384	0.0189	-1.6513	0.0001	-2.5811	1.96E-23	Y
1	8095	hypothetical protein VspiD_30155 [Verruc...	3.00E-05	-1.2486	0.5077	-1.2556	0.2471	-1.3504	0.0005	-2.4498	3.44E-11	Y
1	11088	f-box protein family-like protein	1.00E-04	-1.2353	0.0954	-1.2478	0.2247	-1.2592	0.1326	-1.8695	5.15E-06	Y
1	11584	atp-dependent-binding subunit	1.00E-40	-1.2326	0.0441	-1.0504	0.5749	-1.0472	0.5985	-1.799	7.15E-06	Y
1	6758	ankyrin repeat	1.00E-12	-1.1818	0.3072	-1.9483	0.1522	-1.2461	0.1775	-2.8656	3.28E-07	Y
1	4279	acid phosphatase	1.00E-31	-1.157	0.42	-1.0006	0.9899	-2.025	1.67E-06	-1.6078	4.22E-18	Y
1	10766	SIR2-like histone deacetylase [Zea mays]	6.00E-48	-1.153	0.267	-1.097	0.6382	-1.4058	0.0282	-1.9867	6.16E-06	Y
1	4383	protein kinase domain containing protein	1.00E-13	-1.1456	0.6832	-1.6486	0.1276	-1.25	0.2402	-2.4513	3.75E-05	Y
1	11186	zinc finger protein	1.00E-04	-1.1267	0.4613	-1.2864	0.3605	-1.5159	0.009	-2.2716	9.62E-05	N
1	6842	asparagine synthase (glutamine-hydrolyzing)	1.00E-23	-1.1109	0.475	-1.1579	0.4011	-1.1215	0.2943	-1.8223	4.32E-05	Y
1	10139	aldehyde oxidase	1.00E-04	-1.107	0.1309	-1.1413	0.3123	-1.3168	0.0003	-1.7478	4.27E-08	Y
1	8022	ribosomal protein s18	1.00E-06	-1.0982	0.4043	-1.3751	0.006	-1.1496	0.3646	-1.7984	1.05E-05	Y
1	8382	glucose-repressible alcohol dehydrogenase tran	1.00E-14	-1.0945	0.2233	-1.1137	0.1649	-1.5657	4.72E-10	-1.7352	6.40E-07	N
1	7441	PREDICTED: similar to KIAA1127 protein [C	5.00E-06	-1.0915	0.1231	-1.2461	0.0079	-1.2678	0.0954	-1.7353	1.99E-20	Y
1	6015	protein	1.00E-12	-1.0824	0.6449	-1.4266	0.088	-1.4609	0.0364	-2.1218	5.06E-05	Y
1	1939	flavodoxin	1.00E-124	-1.0515	0.7	-1.391	0.0462	-1.3866	0.0107	-1.8681	7.95E-05	Y
1	3235	putative DMI1 protein [Oryza sativa (japoni...	6.00E-12	-1.0412	0.5243	-1.1817	0.0178	-1.3912	2.63E-08	-1.8359	8.64E-12	Y
1	5745	---NA---	1.00E-10	-1.0339	0.7299	-1.0004	0.9973	-1.4239	0.002	-1.7235	6.72E-09	Y
1	5605	3-phosphoadenosine-5-phosphosulfate reductas	1.00E-36	-1.0256	0.8291	-1.1694	0.2838	-1.5132	0.0018	-1.9033	5.50E-07	N
1	6670	rhomboid family protein	1.00E-07	-1.0075	0.9533	-1.3693	0.1155	-1.4513	0.0034	-2.2732	2.41E-08	Y
1	1654	chloroplast light harvesting protein isoform 4	1.00E-74	1.0104	0.9004	-1.5006	7.80E-05	-2.8988	0.009	-2.1841	2.53E-20	N
1	11041	pabp-dependent polynuclease 3 isoform 1	1.00E-06	1.0121	0.8837	-1.486	0.0287	1.0145	0.8674	-1.7204	3.90E-06	Y
1	11115	nitrite extrusion protein 2	1.00E-99	1.0169	0.9088	-1.5138	0.0103	-1.5038	4.11E-05	-1.9361	3.83E-14	Y
1	642	hypothetical protein P9211_15381 [Proch...	1.00E-21	1.0257	0.7742	1.1958	0.0765	-1.4427	0.0002	-1.7377	1.95E-13	N
1	5516	sphingomyelinase c	1.00E-11	1.0282	0.7829	-1.2	0.1904	-1.4119	0.0346	-1.8617	1.71E-08	Y
1	5092	Kynurenine 3-monooxygenase and related flav	6.00E-47	1.0397	0.6037	-1.0921	0.7301	-1.7964	3.40E-08	-1.7235	3.41E-05	Y
1	6521	acylamide amidohydrolase [Marinobacter aqu..	1.00E-102	1.0438	0.7388	-1.3386	0.0435	-1.708	2.85E-05	-2.17	2.12E-08	Y
1	1685	S-adenosyl-L-methionine synthetase 2 [Catha..	7.00E-21	1.0755	0.5505	1.142	0.4582	-1.5377	0.0347	-3.3282	1.28E-06	Y
1	5264	thioredoxin domain-containing protein 5 precu	1.00E-46	1.0925	0.4198	-1.3483	0.0051	-1.5662	9.38E-05	-1.7778	1.34E-07	N
1	10787	clp protease atp binding subunit	1.00E-75	1.1075	0.4226	1.1021	0.6473	-1.1012	0.727	-2.6674	9.93E-14	Y
1	11346	phosphodiesterasecgmp-delta	1.00E-39	1.1257	0.3276	1.0392	0.8324	-1.8217	7.82E-05	-1.3459	0.1584	Y
1	9851	ribosomal protein l16	1.00E-31	1.1284	0.309	1.2328	0.2241	-1.2539	0.1908	-2.9833	8.38E-06	N
1	2180	photosystem II core 32 kDa protein [Gymnodi.	1.00E-142	1.1373	0.4425	1.3125	0.1463	-1.0336	0.8774	-2.8007	1.35E-10	Y
1	10102	aarf domain containing kinase 1	1.00E-21	1.1602	0.1703	-1.4995	8.10E-05	-1.032	0.7715	-1.787	2.44E-05	Y
1	11045	preprotein translocase subunit	1.00E-63	1.2203	0.2504	1.2707	0.3348	-1.0792	0.7433	-3.2744	1.47E-16	Y
1	10543	super cysteine rich protein; SCRP [Homo sapi..	4.00E-10	1.2334	0.11	-1.1287	0.5348	-1.1611	0.2885	-1.866	7.10E-06	N

1	4999	ribosomal protein s4	1.00E-25	1.4041	0.0111	1.2584	0.1982	-1.0272	0.8943	-2.5155	1.93E-14	Y
1	5265	appr-1-p processing enzyme family prote...	2.00E-06	1.4454	0.0076	-1.1033	0.5967	-1.737	3.36E-06	-1.5842	0.0007	Y
2	7526	30S ribosomal protein S4 [Microcystis a...	9.00E-05	-1.424	0.001	-1.4388	0.0958	2.2935	2.45E-05	1.3942	0.1415	N
2	4692	cog0561: hydrolases of the had superfamily	1.00E-117	-1.2397	0.0877	-1.7762	3.78E-05	-1.2009	0.3193	-1.5769	0.0055	Y
2	95	proliferating cell nuclear antigen [Karenia ...	1.00E-125	-1.1197	0.5548	-1.0782	0.7786	1.7636	0.0122	2.9982	7.89E-08	N
2	4982	zinccchc domain containingisoform cra_a	1.00E-10	-1.1022	0.5368	-1.0209	0.8486	1.2663	0.0423	1.9067	2.20E-05	Y
2	7262	kinesin motor domain containing protein	1.00E-35	-1.095	0.4748	1.0115	0.908	1.9125	7.65E-19	1.2838	0.0006	Y
2	5658	predicted protein [Chlamydomonas reinha...	1.00E-06	-1.0273	0.8327	1.2097	0.097	1.821	1.04E-07	1.2925	0.0444	Y
2	4928	pentatricopeptiderepeat-containing protein	1.00E-18	-1.0266	0.8229	1.3489	0.0356	2.0998	6.20E-08	1.9979	0.0003	N
2	2185	dead deah box helicase family protein	1.00E-07	-1.024	0.8581	1.0744	0.7589	1.7599	0.0005	2.4241	4.94E-07	Y
2	3070	ran binding-like protein 1 [Babesia divergens]	1.00E-32	-1.0136	0.9135	1.2581	0.0935	1.8534	1.73E-06	1.3972	0.022	Y
2	9074	phenolpthiocerol synthesis type-i polyketide sy	1.00E-27	1.0047	0.979	1.0989	0.4053	-1.0341	0.8475	1.7732	2.46E-06	Y
2	7702	possible esterase lipoprotein lpqc	1.00E-11	1.0052	0.9674	-1.0294	0.7593	1.2859	0.0189	1.8625	1.16E-08	N
2	7813	FHA domain protein, putative [Plasmodium ch	8.00E-06	1.0103	0.8916	1.0529	0.712	1.6742	0.0001	1.8465	5.10E-07	Y
2	1715	tubulin alpha chain	1.00E-66	1.0321	0.7721	1.3473	0.213	2.3065	1.42E-05	2.5014	1.19E-07	Y
2	3987	phosphatidylinositol-4-phosphate 5-kinase	1.00E-16	1.0438	0.6887	1.4017	0.0085	1.3229	0.0205	1.812	6.12E-07	Y
2	6608	KH domain containing protein [Brugia ma...	5.00E-06	1.0496	0.7207	1.3027	0.1963	1.4102	0.0581	1.9063	6.49E-05	Y
2	10706	hypoxia-inducible factoralpha subunit inhibitor	1.00E-04	1.0547	0.5435	1.0528	0.7167	1.2998	0.1173	1.7191	6.36E-24	Y
2	11587	putative insulin degrading enzyme [Oryza s...	8.00E-11	1.0778	0.5231	1.2914	0.0899	1.8577	5.62E-06	1.4914	0.0016	Y
2	10554	vic family transporter: potassium ionpotassium	1.00E-05	1.08	0.2275	1.177	0.119	1.921	3.42E-15	1.5342	8.93E-07	Y
2	3582	PREDICTED: similar to DEAD (Asp-Glu-Ala-	3.00E-45	1.0825	0.5793	1.1456	0.3408	1.3796	0.0144	1.7189	2.73E-05	Y
2	5197	transcription termination factor rho	1.00E-04	1.0856	0.1696	-1.0309	0.8637	1.6388	1.04E-13	1.7255	2.19E-16	Y
2	8734	fragile x mentalautosomal homolog 2	1.00E-06	1.0881	0.335	1.3274	0.2682	2.311	1.50E-14	2.3173	2.51E-12	Y
2	8774	h+-ppase family transporter: proton	1.00E-24	1.0924	0.4902	1.4323	0.0064	1.7994	1.11E-06	1.6974	0.0025	Y
2	5673	3-ketoacyl-coa thiolase-like protein	1.00E-66	1.0945	0.1636	1.2876	0.0017	1.3853	1.54E-08	1.7483	1.65E-15	Y
2	3299	protein with PPR repeats [Chlamydomonas...	2.00E-21	1.0967	0.3436	-1.053	0.6507	1.2993	0.0095	1.7434	3.72E-09	Y
2	6049	kinesin-like calmodulin-binding protein	1.00E-40	1.1064	0.1758	1.0714	0.6813	1.6408	8.58E-10	1.9901	5.40E-14	N
2	9232	ef hand domain protein	1.00E-07	1.1307	0.2807	1.4937	0.0007	1.8578	3.71E-07	1.3084	0.1508	Y
2	11662	serine threonine-specific protein phosphatase	1.00E-24	1.1536	0.2995	1.422	0.0324	2.0454	2.05E-05	1.5811	0.0549	Y
2	11001	armadillo repeat containing 3	1.00E-07	1.1569	0.0373	1.2321	0.0073	1.306	0.0012	1.7146	8.16E-06	Y
2	2272	---NA---	1.00E-04	1.1615	0.1444	-1.0377	0.8012	1.9695	2.55E-09	2.5565	2.49E-15	N
2	4920	bnp_lacmubradykinin-potentiating and c-type r	1.00E-04	1.1769	0.1326	1.2774	0.0765	1.3614	0.0199	1.7293	1.82E-05	Y
2	7842	regulator of nonsense transcripts-related protei	1.00E-14	1.1792	0.1758	1.1123	0.4382	1.3443	0.0158	1.7587	2.33E-05	Y
2	4879	aspartyl proteinase (eimepsin) [Eimeria ten...	1.00E-47	1.1908	0.0417	1.2168	0.0168	1.2515	0.0411	1.764	9.16E-12	Y
2	6533	s-adenosyl-methyltransferase	1.00E-11	1.1945	0.1401	1.3429	0.021	1.6708	6.30E-05	1.8919	1.49E-05	Y
2	11417	dead (asp-glu-ala-asp) box polypeptide 51	1.00E-12	1.1983	0.147	1.2923	0.0503	1.8251	1.47E-05	1.5151	0.0117	N
2	8698	cog3321: polyketide synthase modules and rela	1.00E-12	1.1998	0.3547	1.1796	0.2864	-1.0073	0.9765	2.2517	1.12E-05	Y
2	5142	---NA---	1.00E-18	1.2076	0.0889	1.2067	0.1697	1.4109	0.0055	1.8149	2.49E-05	Y
2	858	conserved hypothetical protein [Plasmodium ...	1.00E-08	1.2139	0.0975	1.2594	0.0729	1.4551	0.0015	1.927	8.86E-08	Y
2	11642	g-protein beta wd-40 repeat	1.00E-06	1.2152	0.2219	1.4656	0.0264	1.4937	0.0066	1.9044	2.71E-05	Y

2	6256	atp-dependent protease	1.00E-123	1.223	0.0304	1.3682	0.0069	1.8731	7.25E-10	1.6431	1.92E-06	Y
2	6631	polyketide synthase	1.00E-26	1.2257	0.2778	1.2404	0.1439	1.0853	0.7868	2.0131	1.07E-05	Y
2	6125	circumsporozoite protein	1.00E-04	1.2418	0.0237	1.3429	0.0147	1.7705	1.30E-08	1.2996	0.0295	Y
2	8982	putative TPR (tetratricopeptide repeat) doma...	4.00E-06	1.2469	0.1265	1.3494	0.0278	1.2235	0.1311	1.8778	3.49E-05	Y
2	927	---NA---	1.00E-04	1.2609	0.002	-1.1247	0.5495	1.1146	0.1444	1.9141	6.72E-23	Y
2	7086	mitochondrial creatine kinase CK2 [Tethya au...	5.00E-40	1.3129	0.0281	1.5057	0.0078	1.2957	0.061	1.7984	2.47E-06	Y
2	1365	similar to ubiquitin specific protease ...	3.00E-18	1.3355	0.0151	1.4016	0.035	1.2737	0.0283	1.878	3.29E-08	Y
2	5246	cold shock protein	1.00E-05	1.3382	0.0331	1.2847	0.2215	2.0013	1.09E-05	1.6091	0.004	Y
2	10204	PREDICTED: similar to EH domain binding pr	9.00E-08	1.3567	0.0371	1.0984	0.5586	1.6734	0.0002	2.1354	1.38E-08	Y
2	3880	E-selectin [Equus caballus] >gi|38503140|sp|...	7.00E-05	1.3612	0.0202	1.5106	0.0016	1.602	0.0007	2.1708	8.59E-07	Y
2	9821	hexosaminidase b	1.00E-50	1.4301	0.0001	1.4712	1.15E-05	1.4288	1.04E-06	1.8214	6.02E-08	Y
2	5137	Kynurenine 3-monooxygenase and related flav.	8.00E-42	1.5052	8.95E-09	1.7774	2.90E-05	1.1827	0.0108	1.203	0.2352	Y
2	9868	expressed lipoprotein	1.00E-09	1.6371	9.17E-10	1.0982	0.458	1.806	1.45E-05	1.3963	0.0012	Y
2	7544	cellulase (glycosyl hydrolase family 5)	1.00E-39	1.6789	0.0002	1.2441	0.0691	2.1964	1.25E-06	1.7834	2.08E-08	Y
2	9243	translation elongation factor ef-subunit	1.00E-43	1.7559	0.0275	1.0752	0.7741	2.9301	2.14E-05	1.8158	0.02	Y
3	8322	60s ribosomal protein l2	1.00E-57	-3.3706	0.0036	-2.2515	8.86E-07	-1.5673	0.1819	-2.9634	5.15E-06	Y
3	11683	sec-independent protein translocase-like p...	1.00E-13	-1.6305	5.72E-07	-2.0377	0.0113	-1.0976	0.5335	-2.789	3.49E-15	N
3	11273	potassium channel beta subunit	1.00E-28	-1.5184	0.2533	-1.318	0.3912	-1.8487	0.004	-4.7427	2.44E-08	Y
3	3209	flagellar calcium-binding protein	1.00E-15	-1.471	0.0603	-1.9346	0.0091	-1.693	0.0113	-3.2607	2.38E-07	Y
3	8932	Photosystem I P700 chlorophyll A apopr...	7.00E-67	-1.4491	0.0036	-1.4849	0.0367	-1.6731	0.0346	-5.1932	1.74E-15	N
3	1331	universal minicircle sequence binding protein	1.00E-13	-1.4274	0.1771	-1.5227	0.1783	-1.329	0.0222	-5.2198	4.43E-06	Y
3	11815	atp synthase cf0 a subunit	1.00E-11	-1.361	0.0075	-1.5506	0.0777	-1.213	0.3742	-3.0549	5.26E-06	N
3	11850	P700 chlorophyll a apoprotein [Gymnodinium	1.00E-106	-1.339	0.0315	-1.2776	0.4096	-1.6952	0.0192	-9.6368	2.44E-26	N
3	5329	nle1 protein	1.00E-54	-1.2943	0.2616	-1.2161	0.5827	-1.8874	0.0204	-3.8035	1.87E-05	Y
3	9310	cleavage and polyadenylation specificity factor	1.00E-63	-1.2307	0.2012	-1.4928	0.2252	-1.4249	0.0241	-2.9332	3.78E-06	N
3	5044	26s proteasome atpase subunit	1.00E-85	-1.1624	0.5916	-1.7886	0.189	-1.3885	0.2608	-7.9375	9.29E-06	Y
3	4572	photosystem i p700 chlorophyll a apoprotein a2	1.00E-56	-1.1585	0.3719	1.0134	0.9421	-1.4192	0.0393	-4.0947	6.93E-14	Y
3	3095	ubiquitin-ribosomal protein fusion S27a [Can...	7.00E-34	-1.1122	0.633	-1.6359	0.2769	-1.7384	0.0189	-3.7719	3.09E-05	Y
3	6307	magnesiumatpase subunit i	1.00E-90	-1.1107	0.4288	-1.0902	0.7002	-1.4847	0.0378	-3.786	2.39E-08	Y
3	9488	photosystem ii 47 kda protein	1.00E-156	-1.0807	0.4981	-1.1176	0.585	-1.481	0.0148	-6.5211	3.11E-38	Y
3	10994	photosystem i p700 chlorophyll a apoprotein a2	1.00E-109	-1.0767	0.6503	-1.0528	0.8374	-1.2092	0.2657	-6.4644	3.32E-29	Y
3	6284	molecular chaperone DnaK [Anabaena variabi.	3.00E-49	-1.0624	0.676	-1.0682	0.7388	-1.3042	0.2313	-3.8496	3.43E-22	Y
3	10078	atp synthasealpha subunit	1.00E-19	-1.0481	0.623	-1.1324	0.5113	-1.2495	0.2712	-3.6489	9.29E-33	Y
3	9479	photosystem ii 44 kda protein	1.00E-170	-1.0291	0.793	-1.0105	0.9624	-1.4685	0.0084	-4.9234	7.72E-28	N
3	4197	ribulose-1,5-bisphosphate carboxyl...	1.00E-104	-1.0093	0.9454	1.0424	0.7777	-1.1721	0.3047	-3.4399	9.77E-29	Y
3	11444	ribosomal protein l5	1.00E-25	-1.0064	0.9425	-1.2276	0.5271	-1.8039	0.0029	-6.2334	3.50E-38	Y
3	10035	photosystem ii d2 protein	1.00E-110	1.0212	0.8809	1.0338	0.8739	-1.2199	0.1983	-3.8087	6.47E-13	Y
3	10308	50s ribosomal protein l22	1.00E-18	1.0988	0.4334	-1.0366	0.8871	-1.4715	0.1083	-4.6436	4.30E-33	Y
4	1841	unnamed protein product [Ostreococcus tauri]	1.00E-37	-1.4195	4.07E-05	-1.9678	2.56E-09	-1.3643	0.0006	-1.4096	4.26E-05	Y
4	10146	60s ribosomal protein l21	1.00E-66	-1.3881	0.0165	-1.5518	0.0037	-1.8479	1.50E-06	-2.031	2.02E-13	Y

4	526	alpha- alpha-d-galactoside	1.00E-18	-1.3738	0.0001	-1.2942	0.0126	-1.7616	2.51E-10	-1.2457	0.0011	Y
4	11107	required with rad23 for duplication of the spind	1.00E-10	-1.3711	6.01E-07	-1.7615	2.60E-11	-1.0752	0.3688	-1.3643	2.81E-06	Y
4	5399	hypothetical protein XfasA02001193 [Xyle...	1.00E-05	-1.3648	0.0009	-2.0317	4.96E-06	-2.199	4.72E-15	-2.2124	1.23E-08	Y
4	3653	ribosomal protein s2	1.00E-15	-1.3327	0.0009	-1.3051	0.0223	-2.0633	4.20E-05	-2.207	0.0004	N
4	8693	Pepsin II-1 precursor (Pepsin A) >gi|...	8.00E-07	-1.325	0.0279	-1.3383	0.1167	-1.747	8.26E-05	-2.1527	3.15E-05	Y
4	4562	oligosaccharyl transferase	1.00E-51	-1.3146	0.011	-1.3174	0.1637	-1.8851	5.89E-12	-2.4232	5.50E-13	Y
4	5939	PREDICTED: similar to tensin [Gallus gallus]	2.00E-05	-1.2936	0.1604	-1.0945	0.764	-1.7958	0.0098	-2.3743	9.47E-05	Y
4	9789	coae_bachd dephospho-kinase (dephosphocoen	1.00E-23	-1.2834	0.0067	-1.7616	0.0008	-1.4443	0.007	-1.8779	1.78E-14	Y
4	113	glutamic acid-rich protein cnbl1500	1.00E-07	-1.2691	0.0253	-1.3601	0.0035	-1.5214	0.0023	-1.9115	2.58E-08	Y
4	6849	similar to repetin [Rattus norvegicus]	2.00E-06	-1.2685	0.0144	-1.3189	0.0262	-2.2173	2.89E-06	-1.5935	0.0835	Y
4	10088	chitobiase-like [Hyperamoeba dachnaya]	4.00E-13	-1.2661	0.0031	-1.3866	0.0644	-1.7992	8.61E-10	-1.7289	3.38E-16	Y
4	4832	ion transport protein	1.00E-04	-1.2584	0.0015	-1.25	0.0172	-1.7302	9.51E-12	-1.5259	6.22E-05	Y
4	1695	chloroplast light harvesting protein isoform 12	1.00E-08	-1.2551	0.0841	-1.2819	0.03	-1.6063	0.0092	-1.7407	8.80E-06	Y
4	8535	dynein light chainflagellar outer	1.00E-16	-1.234	0.0258	-1.5541	0.0072	-1.3641	0.002	-1.7251	1.67E-05	Y
4	455	cg6282-isoform a	1.00E-13	-1.2187	0.0385	-1.7316	2.78E-09	-1.2758	0.0229	-1.2526	0.0125	Y
4	8640	40S ribosomal protein S5 [Nicotiana plumbag..	6.00E-68	-1.2142	0.1068	-1.2116	0.2221	-2.1803	9.78E-06	-2.272	2.39E-09	Y
4	315	carboxypeptidase type III [Theobroma cacao]	1.00E-26	-1.2128	0.2329	-1.3862	0.076	-2.3067	6.58E-09	-2.4946	6.22E-12	Y
4	2876	dehydrogenase MVIM-sugar aminotransferase	3.00E-13	-1.2073	0.2074	-1.1797	0.2216	-1.9325	5.10E-06	-1.7071	1.45E-08	Y
4	1236	major basic nuclear protein [Gyrodinium gala..	4.00E-28	-1.206	0.0888	-1.7229	1.88E-06	-1.7658	2.63E-11	-1.7995	6.38E-10	Y
4	2840	s-adenosylmethionine synthetase	1.00E-14	-1.1979	0.1341	-1.7507	5.16E-06	-1.5475	5.06E-05	-1.6882	3.00E-05	Y
4	2043	---NA---	1.00E-36	-1.1881	0.0717	-1.6059	2.78E-09	-1.6517	7.48E-07	-2.0483	1.87E-08	Y
4	3789	60s acidic ribosomal protein p0	1.00E-17	-1.1853	0.0381	-1.4183	0.0003	-1.7889	3.20E-10	-1.7303	7.72E-09	N
4	10160	d-amino acid dehydrogenase small subunit	1.00E-07	-1.1706	0.2053	-1.1889	0.4498	-2.1492	1.18E-17	-2.3784	1.64E-05	Y
4	7305	Non-ribosomal peptide synthetase modules a...	2.00E-06	-1.1696	0.0098	-1.2289	0.0288	-1.6493	1.18E-13	-2.6047	8.19E-24	N
4	6089	MPBQ/MSBQ transferase cyanobacterial type	8.00E-07	-1.1623	0.0251	-1.1925	0.0231	-1.8187	0.0002	-2.0906	1.06E-18	N
4	9889	pentapeptide protein	1.00E-10	-1.1531	0.0328	-1.1731	0.1146	-1.7439	1.80E-18	-1.8431	2.13E-20	Y
4	3120	beta-D-mannosidase [Thermobifida fusca]	2.00E-11	-1.1508	0.2499	-1.2797	0.1755	-1.7048	4.07E-05	-2.0538	5.14E-08	N
4	3276	protein with PPR repeats [Chlamydomonas...	5.00E-19	-1.1296	0.1225	-1.3109	0.0906	-1.9596	1.35E-13	-2.5441	6.33E-09	Y
4	6532	glycosylgroup 2 family protein domain protein	1.00E-09	-1.1247	0.2988	-1.1636	0.1963	-1.751	1.12E-05	-1.762	1.14E-05	Y
4	9470	peptidyl-prolyl cis-trans isomerase	1.00E-04	-1.1227	0.2954	-1.1883	0.1489	-1.7619	5.72E-07	-1.2767	0.1253	Y
4	551	eukaryotic translation initiation factor 3 subuni	1.00E-32	-1.1223	0.1855	-1.2848	0.0548	-1.7016	2.41E-10	-1.5261	0.0013	Y
4	6095	ankyrin repeat-containing protein 2	1.00E-06	-1.1201	0.2664	-1.7118	1.33E-13	-2.0271	3.45E-07	-1.1929	0.015	Y
4	10192	---NA---	1.00E-05	-1.1196	0.3242	1.0191	0.8702	-1.6904	2.28E-06	-1.7633	5.97E-07	Y
4	9714	phospholipase carboxylesterase family protein	1.00E-08	-1.1142	0.1614	-1.3275	0.0013	-1.7452	6.31E-08	-1.573	5.16E-08	Y
4	6531	methyltransferase type 11	1.00E-28	-1.092	0.2507	-1.3254	0.0117	-1.9948	7.53E-10	-2.0532	4.28E-14	N
4	6566	calrectulin [Heterocapsa triquetra]	6.00E-30	-1.088	0.4532	-1.1337	0.3782	-2.2172	1.25E-06	-1.9439	2.47E-07	N
4	7361	O-methyltransferase [Chlamydomonas rein...	2.00E-06	-1.0746	0.5483	1.0833	0.5033	-2.0851	6.70E-07	-1.8283	1.68E-05	Y
4	5373	predicted protein of CLR family [Chlamy...	3.00E-07	-1.0548	0.6046	-1.0481	0.7373	-1.8831	2.27E-21	-1.5817	0.001	Y
4	5449	slingshot homolog 2	1.00E-13	-1.051	0.5544	-1.0311	0.7695	-1.7313	3.31E-08	-1.1628	0.152	Y
4	3460	multiprotein bridging factor type 1	1.00E-07	-1.0024	0.9796	-1.3761	0.0004	-1.8	1.08E-10	-2.2322	7.07E-07	Y

4	6523	tpr repeat-containing protein	1.00E-05	1.0038	0.9647	-1.0564	0.5021	-2.0385	8.34E-27	-2.4497	1.85E-16	N
5	3307	pentatricopeptiderepeat-containing protein	1.00E-24	1.1849	0.544	2.0568	2.24E-05	1.7376	0.0002	1.4708	0.0224	N
5	3280	pentatricopeptiderepeat-containing protein	1.00E-21	1.394	4.05E-06	1.8511	7.89E-19	1.5547	8.08E-06	1.0867	0.5247	N
5	3289	pentatrichopeptide repeatprotein	1.00E-06	1.4232	0.0175	4.321	3.39E-33	3.3151	1.37E-06	1.7756	1.65E-05	N
5	3256	pentatricopeptiderepeat-containing protein	1.00E-11	1.4287	0.0038	4.5545	3.14E-28	1.9263	4.25E-07	1.5343	0.0023	Y
5	2853	protein with PPR repeats [Chlamydomonas...	2.00E-22	1.4383	2.46E-05	1.9087	1.63E-11	1.1662	0.2652	1.1608	0.52	N
5	3260	pentatricopeptiderepeat-containing protein	1.00E-23	1.5284	9.69E-05	2.0248	3.83E-09	1.2574	0.0219	1.392	0.0011	Y
5	3262	ENSANGP00000029120 [Anopheles gambiae	1.00E-04	1.5345	0.0011	2.1645	5.96E-07	1.3106	0.05	1.2015	0.1739	N
5	3293	pentatricopeptiderepeat-containing protein	1.00E-17	1.5398	0.0054	2.337	1.11E-06	1.3996	0.0193	1.1964	0.3741	N
5	3255	pentatricopeptiderepeat-containing protein	1.00E-24	1.5636	0.0039	2.0194	5.10E-06	1.8365	0.0003	1.6439	0.004	N
5	3295	PUTATIVE PPR REPEATS CONTAINING P	5.00E-06	1.6501	0.0007	2.245	3.01E-07	1.1374	0.3846	1.252	0.1773	N
5	3281	pentatricopeptiderepeat-containing protein	1.00E-04	1.6525	1.66E-07	2.1872	9.61E-19	1.723	5.55E-08	1.6507	4.28E-06	N
5	3291	pentatricopeptiderepeat-containing protein	1.00E-33	1.6852	0.0007	2.2007	2.51E-07	1.7922	0.0006	1.6373	0.005	N
5	3292	pentatricopeptiderepeat-containing protein	1.00E-28	1.7371	4.19E-05	1.9105	0.0008	1.7917	0.0001	1.7642	0.0003	N
5	3279	pentatricopeptiderepeat-containing protein	1.00E-29	1.7655	0.0004	2.1665	2.12E-06	1.8476	0.0003	1.6616	0.0097	N
5	3973	PUTATIVE PPR REPEATS CONTAINING P	2.00E-26	1.7706	0.0004	2.4947	4.24E-06	1.4625	0.0167	1.3335	0.0988	N
5	3257	pentatricopeptiderepeat-containing protein	1.00E-24	1.7901	6.94E-05	2.5319	2.03E-09	1.4583	0.0054	1.1142	0.549	N
5	5093	rna-binding protein	1.00E-22	1.7984	2.06E-06	2.0652	2.07E-05	1.4684	0.0021	1.549	0.0079	Y
5	3258	pentatricopeptiderepeat-containing protein	1.00E-36	1.8186	0.0013	2.4185	3.01E-06	1.8141	0.0033	1.654	0.0188	N
5	3266	pentatricopeptide (ISS) [Ostreococcus tauri]	4.00E-06	1.8908	0.0005	2.3767	4.54E-05	1.636	0.002	1.4635	0.0625	N
5	3261	pentatricopeptiderepeat-containing protein	1.00E-15	1.9836	5.28E-11	2.7399	3.32E-22	1.7452	7.71E-06	1.3763	0.0498	Y
5	3254	PUTATIVE PPR REPEATS CONTAINING P	8.00E-16	2.1586	1.14E-05	2.7198	9.14E-07	1.9265	0.0004	1.6161	0.0245	N
5	3278	PUTATIVE PPR REPEATS CONTAINING P	5.00E-29	2.4145	2.26E-08	3.267	5.41E-11	2.0329	5.05E-06	1.8412	0.0009	N
5	183	pentatrichopeptide repeatprotein	1.00E-06	3.0183	2.12E-13	6.914	6.19E-32	3.2022	1.24E-14	2.8366	1.02E-09	N
5	1651	nuclear pore complex component (sc seh1)	1.00E-11	3.2888	1.82E-07	1.3064	0.2588	2.1697	0.0058	1.4537	0.2464	Y
5	3271	pentatricopeptiderepeat-containing protein	1.00E-11	3.4207	5.58E-08	4.3232	2.91E-13	2.1193	0.0001	3.388	2.19E-08	N

[a]e-value of top BLASTx hit

[b]Unique to P-addition trend set

The time points at which a probe qualified for inclusion in the trend set are indicated in red (up) or green (down)

FC: fold change
